# The cognitive effects of supplementation with sunflower phosphatidyl serine in healthy children aged 8 to 12 years: a randomized controlled trial

**DOI:** 10.1186/s12937-025-01264-9

**Published:** 2025-11-29

**Authors:** Marina Friling, Philippa A. Jackson, David Kennedy, Fiona Dodd, Ellen Smith, Arava Lavie, Adrian Lopresti, Eran Ivanir, Jonna Jalanka

**Affiliations:** 1 IFF Health, Migdal Haemek, Israel; 2 Brain, Performance and Nutrition Research Centre, Northumbria University, Newcastle upon Tyne, UK; 3 Clinical Research Australia, Perth , Australia; 4 IFF Health & Biosciences, Kantvik, Finland

**Keywords:** Phosphatidylserine, Dietary supplement, Sharp PS^®^ green, Cognitive performance, Healthy children, Visuospatial memory

## Abstract

**Background:**

Supplementation of the diet with phosphatidylserine (PS) is associated with cognitive and neuropsychological benefits in healthy and neuro-compromised adults. It has also been shown to mitigate symptoms of inattention in children with attention-deficit hyperactivity disorder. However, there is little data on the effects of PS in healthy children.

**Objective:**

The aim of this randomized, placebo-controlled clinical trial was to examine the effects of sunflower-derived PS on cognitive performance in healthy, neurotypical children aged 8–12 years.

**Methods:**

Participants received 100 mg of sunflower-derived PS daily in gummy form or a matching placebo for 12 weeks and completed an assessment battery at baseline and after 6 and 12 weeks to monitor changes in cognitive performance, mood, and sleep. Retrospectively registered at Clinicaltrials.gov; NCT05177978

**Results:**

There were no differences in the primary or secondary outcomes in the total cohort. However, in a pre-defined subgroup analysis of children who were selected based on their constant below median performance across the cognitive tasks at baseline, PS-supplementation showed benefit on a visuospatial memory task. The supplementation with 100 mg of Sharp PS green was shown to be safe and well tolerated.

**Conclusion:**

Although there were no differences in the primary and secondary outcomes, the findings suggest that future research should focus on children with below median performance, who are more prone to benefit from PS supplementation.

**Supplementary Information:**

The online version contains supplementary material available at 10.1186/s12937-025-01264-9.

## Introduction

Phosphatidylserine (PS), a structural component of mammalian cell membranes, is an important phospholipid in physiological health. It is enriched in neuronal tissue, wherein PS accounts for 13–14% of total phospholipid content in the human brain [1]. Given this abundance, PS is essential in many neuronal functions, including signaling, differentiation, and survival—activities that require PS to participate in electrostatic interactions and interactions with regulatory domains in major signaling molecules, including protein kinase C and Akt [2, 3].

In addition to *de novo* synthesis from phosphatidylcholine and phosphatidylethanolamine [2], PS can be obtained from the diet, typically via meat and fish consumption. The intake of PS has been declining in the Western population due in part to changing dietary trends and the inherent pathogenicities of specific PS sources. The safety concerns over bovine-derived PS, based on potential contamination by bovine spongiform encephalopathy prions [4], have led to the availability of both soybean- and marine-derived forms as alternatives. Unfortunately, the allergenicity of soybean-derived foods [5] and animal-based products [6] poses issues for those with such allergies, prompting the development of PS from different origins, such as sunflower.

Several clinical trials have been performed on neuro-compromised adults to determine the effects of supplementation of PS on cognitive function. In Alzheimer’s disease, the administration of PS has been associated with improved memory, greater stability in daily functioning, and better mood [7]; improved neuropsychological function [8]; and partially restored neurotransmitter concentrations [9]. As part of a multicomponent supplement, consumption of PS was associated with improved mood, as demonstrated by a reduction in the depression score of elderly patients suffering from major depression [10]. Supplementation with soybean-derived PS has been shown to increase exercise capacity while accelerating muscle recovery [11] and may provide neurological benefits. Notably, reductions in endogenous PS levels have been paralleled by the appearance of neurological conditions, including depression and autism, implicating PS as a potential biomarker of neurodevelopmental disorders [12–14]. Further, as a supplement, PS, whether derived from soybean or sunflower, has been designated as Generally Recognized as Safe (GRAS) by the Food and Drug Administration (FDA) [15, 16]. Specifically, soybean-derived PS has been shown to be safe for human consumption [4, 8, 17]. Thus, PS is a safe supplement with documented benefits in neurological disorders. Thus, PS demonstrates documented benefits as a supporting and maintaining cognitive performance, while remaining distinct from a therapeutic agent.

In contrast, the cognitive impact of PS in healthy, neurotypical individuals is poorly understood. In a recent study, Zhang and colleagues administered a PS/magnesium/vitamin-based supplement to healthy adults. This combination significantly improved clinical memory test scores compared with a placebo, with greater benefits seen in older versus younger participants [18]. The authors concluded that this supplement enhanced memory and cognition in healthy adults but could not identify the specific benefits of PS administered alone. However, as discussed, several randomized controlled trials have demonstrated that PS, delivered in isolation, may be beneficial. In another trial with men with chronic stress, PS was shown to normalize dysregulation in the hypothalamus-pituitary-adrenal axis activity [19] and improved cognitive performance in nondemented elderly subjects [20]. Further, PS improved delayed verbal recall in older adults with mild cognitive impairment [21]. Thus, in the limited available data, PS appears to have positive effects in generally healthy adults.

The cognitive-enhancing effects of PS in healthy children are scarce. In a recent meta-analysis of randomized clinical trials, it was concluded that the administration of PS ameliorated symptoms of inattention in children aged 18 years and younger with attention-deficit hyperactivity disorder (ADHD). In a randomized, placebo-controlled study on healthy high school students aged 17–18 years, PS-fortified milk significantly improved memory [22]. However, whether neurotypical school-aged children benefit cognitively from PS supplementation remains to be determined. Therefore, the objective of this randomized, double-blind, placebo-controlled clinical trial was to investigate the effects of a sunflower-derived PS supplement (Sharp-PS^®^ Green) on cognitive performance in healthy, neurotypical children aged 8–12 years.

## Materials and methods

### Study design and population

**Fig. 1:**
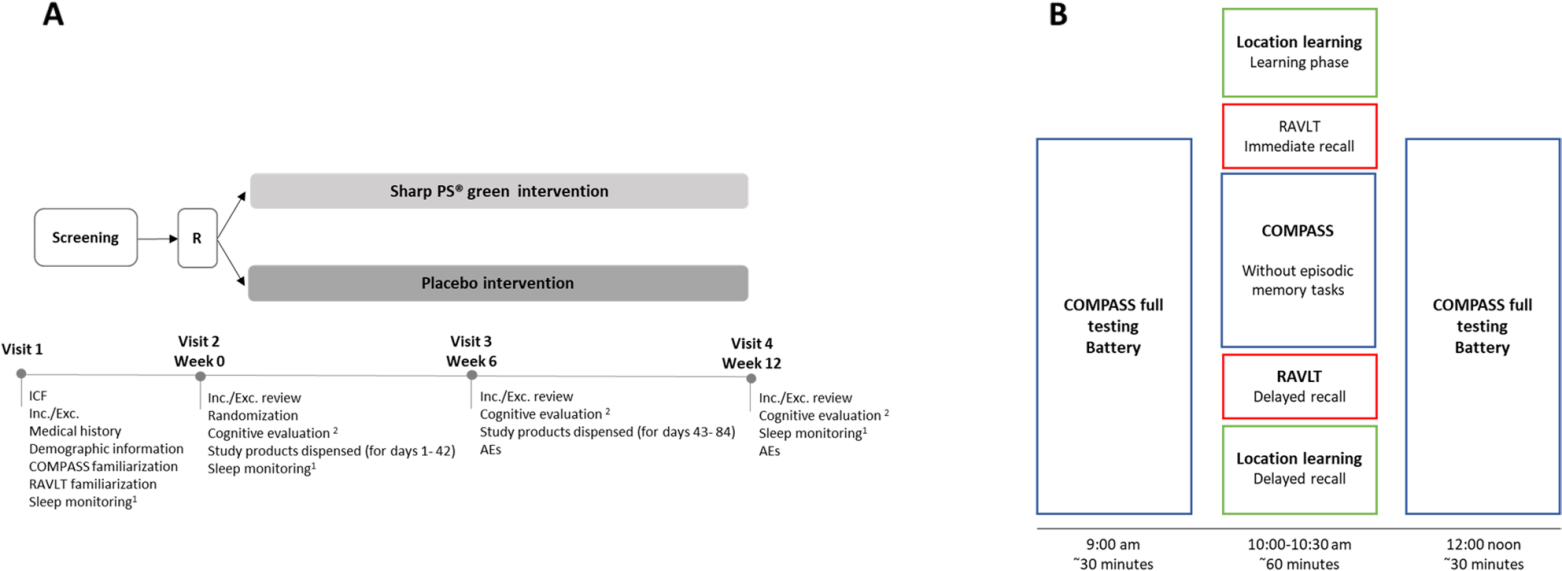
**A** Overview of study design. ^1^ Actigraphy watches were dispensed at visit 1 to 30 participants per group (active and placebo), and sleep was monitored over the 7 days before visits 2 (week 0) and 4 (week 12). ^2^ The cognitive evaluation comprised the COMPASS battery, RAVLT, VAS mood, Parent VAS, SF-CSHQ, and SSR. Abbreviations: R: randomization; ICF: informed consent form; Inc./Exc.: inclusion/exclusion; VAS: visual analogue scale; AEs: adverse events. **B** Overview of testing schedule and timelines on all visits. Participants completed the COMPASS full testing battery at ~ 09:00 and ~ 12:00 (comprising the following individual tasks: VAS mood scale, immediate word recall, simple reaction time, choice reaction time, 4-choice reaction time, arrows flankers, digit vigilance, rapid visual information processing, Stroop task, Corsi blocks task, numeric working memory, 2-back, delayed word recall, delayed picture recognition, and delayed word recognition). The interim assessment (location learning, RAVLT, COMPASS minus episodic memory tasks) had a staggered start to allow for individual RAVLT testing, with participants commencing the battery of cognitive tasks between 10:00 and 10:30

This randomized, double-blind, placebo-controlled, parallel-group, proof-of-concept study (retrospectively registered at Clinicaltrials.gov; NCT05177978) was performed at Northumbria University, UK in accordance with Good Clinical Practice and ethical principles per the World Medical Association (WMA) Declaration of Helsinki (64th WMA General Assembly, Fortaleza, Brazil, October 2013); the EMA Note for Guidance on Good Clinical Practice (CPMP/ICH/135/95 - effective from 17.01.97), commonly referred to as ICH guidelines for GCP; and the laws and regulations that govern clinical research in the UK. The study design is illustrated in Fig. 1.

The protocol (Fig. 1A) consisted of: (1) A telephone screening, comprising informed consent procedures and an eligibility assessment, including the collection of demographic data. (2) A 1-day screening/training visit, in which candidates provided informed consent; underwent the eligibility screen for criteria that could not be collected via the telephone (height, weight); and, if agreeing to participate in the actigraphy study, were assigned actigraphy sleep watches. Training on the computerized tasks and questionnaires was also completed. (3) A baseline visit (visit 2), during which they underwent pre-supplementation assessments. (4) A supplementation period of 12 weeks, in which participants were required to take 2 gummies daily. (5) In-person assessments after 6 and 12 weeks of supplementation.

All in-person assessments were conducted between 9 am and 12.30 pm (Fig. 1B). Participants consumed the same breakfast at home before each visit (cereals or toast), no later than 7.30 am, and abstained from consuming any caffeinated beverage. During each visit, the following cognitive assessments were administered: 9 am: Full COMPASS battery (first assessment), 10:00 to 10:30 am: Computerized Location Learning Task (cLLT) (learning phase), Rey Auditory Verbal Learning Task (RAVLT) (learning phase), attention/working memory tasks from the COMPASS battery (second assessment), delayed recall of Location Learning task, and RAVLT, 12 noon: Full COMPASS battery (third assessment).

### Participants

Eligible participants were children aged 8 to 12 years and in good health, as reported by themselves and their parent/guardian, with a normal sex-related and age-related body mass index (BMI) and who had been speaking English since kindergarten. Potential candidates were recruited through university mailing lists, Facebook groups, advertisements in local newspapers, and school newsletters. The main exclusion criteria were a diagnosis of ADHD, dyslexia, or any neurodevelopmental disorder or learning difficulty; consumption of more than 100 g of high-PS-containing foods per week, following a specific diet routine within 30 days prior to the study; or usage of dietary supplements within the 4 weeks prior to the study. The full list of inclusion and exclusion criteria is provided in the Supplementary Material. All participating children have signed an assent form, and a written informed consent was obtained from all their guardians before study commencement.

### Sample size calculations

Estimating supplementation effect sizes was difficult because no trials have examined the cognitive-enhancing effects of PS in healthy school-aged children. Therefore, a conservative effect size of approximately 0.3 was predicted. Based on G*Power calculations with a standard power of 0.8, an effect size of 0.294 (f = 0.147) is detectable using a sample size of 200 when repeated-measures ANOVA is used for outcomes that are measured 3 times a day. Thus, a total sample of 200 children was planned for enrolment.

### Randomization and blinding

Eligible participants were randomly assigned to receive gummies containing elemental PS (Sharp-PS^®^ Green; active supplementation) or placebo. Investigational products were identical in appearance and taste. Randomization codes were generated by a researcher who was not directly involved in the collection of outcome measures using an online computer generated randomizer (www.randomizer.org), stratified by age and sex.

Supplement labeling, randomization, and unblinding procedures were performed by study personnel who had no further involvement in the study. All other study site personnel, the sponsor representatives, the outcome assessors and statisticians, and study participants were blinded to the assigned study arm. The blinded supplementation code was opened after the statistical study report was finalized and signed.

### Interventions

Participants received 100 mg daily (2 gummies in the morning; taken with food) of sunflower-derived elemental PS orally in gummy form or a matched placebo gummies. The placebo contained identical ingredients except for PS [glucose syrup, sugar, water, citric acid, pectin, trisodium citrate, flavor, and color (black carrot concentrate)]. The supplement and placebo were provided by the sponsor (Frutarom Ltd., Israel; since the start of the trial, Frutarom has merged with International Flavors and Fragrances, New York, NY).

### Outcome measures

Assessments were performed at baseline, week 6, and week 12, with all assessments occurring in person at the Brain Performance and Nutrition Research Centre (BPNRC, UK).

#### Cognitive performance

Participants underwent cognitive assessments at all investigational visits using the Computerised Mental Performance Assessment System (COMPASS) [23–26] and Rey Auditory Verbal Learning Task (RAVLT) [27]. The tasks completed during the RAVLT and COMPASS assessments are detailed in Supplementary Table 1.

The results of the COMPASS cognitive tasks were expressed as composite scores to minimize the impact of variability in individual test scores. The following composite scores were computed: Accuracy of attention, Accuracy of performance, Accuracy of episodic memory, Accuracy of working memory, Speed of attention, Speed of memory, and Speed of performance [28].

#### Visual analogue scale (VAS)

A VAS mood panel was administered via the COMPASS system twice during every visit to evaluate 11 items concerning the child’s mood “at the moment” on a 100-mm scale. Moreover, a VAS was completed by parents at each visit. Parents provided ratings using a 100-mm scale on 5 questions relating to their child’s mood and performance at home and school during the study period. Both VAS panels are included in the Supplementary Material.

#### Sleep quality

Participant guardian-reported sleep quality was assessed using the Children’s Sleep Habits Questionnaire, short form (CSHQ-SF) [29], Children’s Sleep Self-Report (CSSR) [30], and ​​Parent VAS [31]. The CSHQ-SF is a 23-item parent-reported questionnaire designed for school-aged children, yielding both a total score and 6 separate subscales. The CSSR is a 1-week retrospective 26-item questionnaire completed by children aged 7 to 12 years, evaluating sleep constructs similar to the CSHQ-SF, yielding a total score. Both questionnaires were evaluated, and scores were calculated according to common practice.

A subset of participants (30 participants per group) consented to sleep monitoring by an actigraphy watch (ActiGraph wGT3X-BT), which was worn continuously (except while bathing) on the nondominant hand for 7 days before baseline (visit 2) and week 12 (visit 4). The devices collected the following data: sleep onset (first minute that the algorithm recognized as “asleep”), total sleep time (mins), wake after sleep onset (total time that subject was awake after sleep onset; mins), number of awakenings after sleep onset, average awakening time after sleep onset (min), and sleep efficacy, comprising total sleep time/total time in bed (%).

#### Primary and secondary outcome measures

Given the exploratory nature of the study, no single primary endpoint was identified a priori. Instead, a group of outcomes reflecting learning and global cognitive performance after 12 weeks of PS versus placebo were evaluated:

1. Performance measures (COMPASS): speed and accuracy of performance.
2. Attention and memory (COMPASS): speed and accuracy of attention, speed of memory, accuracy of working memory, and accuracy of episodic memory.
3. Location learning (COMPASS): learning index, displacement score, and delayed displacement score.
4. Verbal learning and memory (RAVLT): total learning, immediate recall, delayed recall, and delayed recognition.

The secondary endpoints compared additional aspects of cognitive performance, child’s mood, and overall performance, as well as sleep quality following 6- and 12-week consumption of Sharp-PS^®^ green or placebo (unless specified otherwise):

1. Speed of performance, and accuracy of performance measured by COMPASS system (following 6 weeks of intervention).
2. Speed of attention, accuracy of attention, speed of memory, accuracy of working memory, and accuracy of episodic memory measured by COMPASS system (following 6 weeks of intervention).
3. Learning index, displacement score, and delayed displacement score, measured by COMPASS system using Location Learning task (following 6 weeks of intervention).
4. Total learning, Immediate recall, delayed recall, and delayed recognition measured by the RAVLT (Following 6 weeks of intervention).
5. Individual cognitive task score (immediate word recall, simple reaction time, choice reaction time, 4 choice reaction time, arrows flankers, digit vigilance, rapid visual information processing, Stroop, Corsi blocks, numeric working memory, 2-back, delayed word recall, delayed picture recognition, and delayed word recognition) measured by COMPASS system.
6. Child’s mood measured by mood VAS questionnaire
7. Child’s overall performance measured by parent’s VAS questionnaire.
8. Children’s Sleep Habits Questionnaire-Short Form (SF-CSHQ).
9. Children’s Sleep self-report (CSSR).
10. Sleep duration as reported in a sleep diary (following 12 weeks of intervention).
11. Sleep quality measured by sleep actigraphy device (following 12 weeks of intervention.

### Subgroup analysis of underperformers

**Fig. 2:**
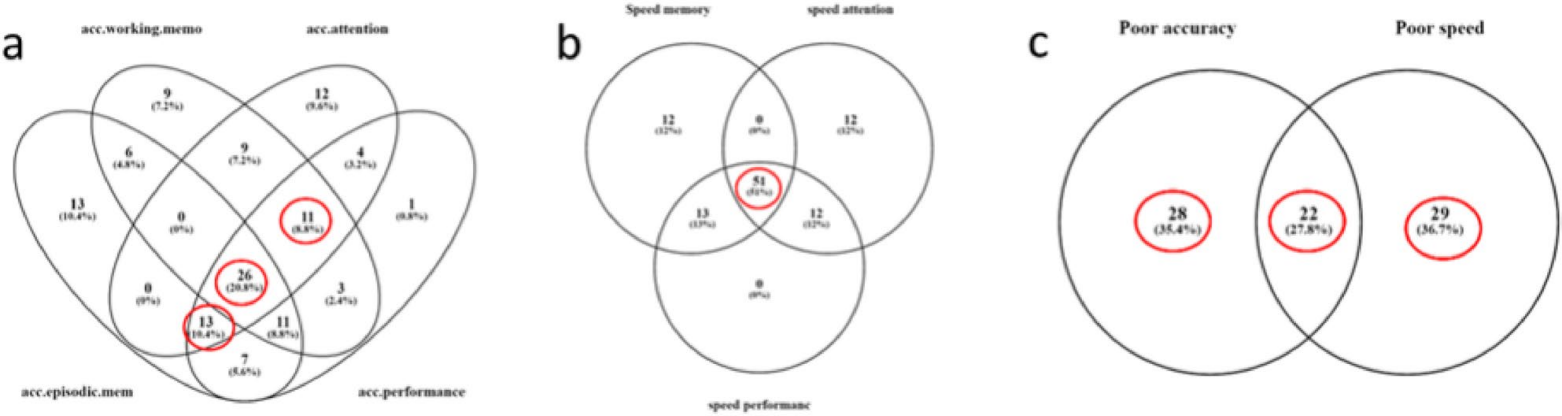
Venn diagram explaining the rationale of selection of the underperforming subgroup. Subjects were performing below median in composite scores for (**A**) accuracy parameters, (**B**) speed parameters, and (**C**) accuracy and speed parameters were selected from PP population into a combined group. The circled numbers represent the number of selected subjects

Changes in the cognitive performance of participants who were classified as ‘underperformers’ were analyzed in a pre-planned subgroup analysis, to test the assumption that efficacy of supplementation may differentiate across certain subsections of the larger cohort. A Venn diagram detailing the selection process of 3 underperforming subgroups is detailed in Supplementary Fig. 1. Briefly, participants who scored below the median for all 7 composite scores were identified. Those who consistently performed below the median in measures for accuracy (n = 50) or speed (n = 51) were selected as separate subgroups (Fig. 2A and B). Because the two underperforming groups overlapped only partially, these underperformers were combined into a single subgroup of interest (Fig. 2C). Participants from the per protocol (PP) population who were not included in the subgroup of underperformers (n = 79) were categorized as typical performers (n = 72) and used as a comparison group.

### Compliance with supplementation and adverse events

Compliance with supplementation included a count of remaining gummies at each active assessment visit and was defined as intake of at least 70% and 80% of gummies after 6 weeks (V3) and 12 weeks (V4), respectively. Any protocol deviations and adverse events were recorded at each visit. Each adverse event was graded according to the following criteria: mild (discomfort but no disruption to normal daily activities), moderate (discomfort that affects normal daily activities), or severe (inability to perform normal daily activities).

### Statistical analysis

The primary and secondary outcomes and subgroup analyses were assessed in all participants who were randomly assigned, completed all study visits, and did not have any major protocol deviations (per protocol population). Baseline and demographic differences between groups were tested using an independent student t-test for continuous variables (age, weight, height, BMI, caffeine consumption, and fruit and vegetable consumption) and chi-square test for categorical variables (school year, biological sex, ethnicity, use of glasses, use of hearing aids, dominant handedness, parent education level, parent employment status, household yearly income, eligibility to free school meals, and dietary habits).

Changes from baseline to weeks 6 and 12 for RAVLT, COMPASS, cLLT, CSHQ, SSR, and VAS scores were analyzed using linear mixed-model analysis with fixed effects for supplementation group (PS vs. placebo), visit (week 6, week 12), assessment time (09:00, 10:00, 12:00), and their interactions. Baseline scores and age (where applicable) were included as covariates. The primary inference was based on the main effect of supplementation (PS vs. placebo) alone.

For actigraphy outcomes, multilevel models were used with supplementation group, night (1–7), and their interaction as fixed effects; pre-supplementation scores were entered as covariates. Participant was included as a random effect where appropriate, and the residual covariance structure was modeled as appropriate.

To control multiplicity for primary outcomes, the Benjamini–Hochberg false discovery rate (FDR) correction was applied. Secondary outcomes and subgroup analyses were unadjusted and interpreted as exploratory. Statistical significance was set at two-tailed *p* < 0.05. Results are reported as means ± SEM with p-values for continuous variables, and n (%) for categorical variables. Estimates represent between-group differences in change from baseline across the study period, unless otherwise specified.

Missing data from the analyzed populations were treated as missing at random. Statistical analyses were performed with SPSS 25.0 (or later) for Windows or R, version 4.2.3.

## Results

### Participant characteristics

**Fig. 3:**
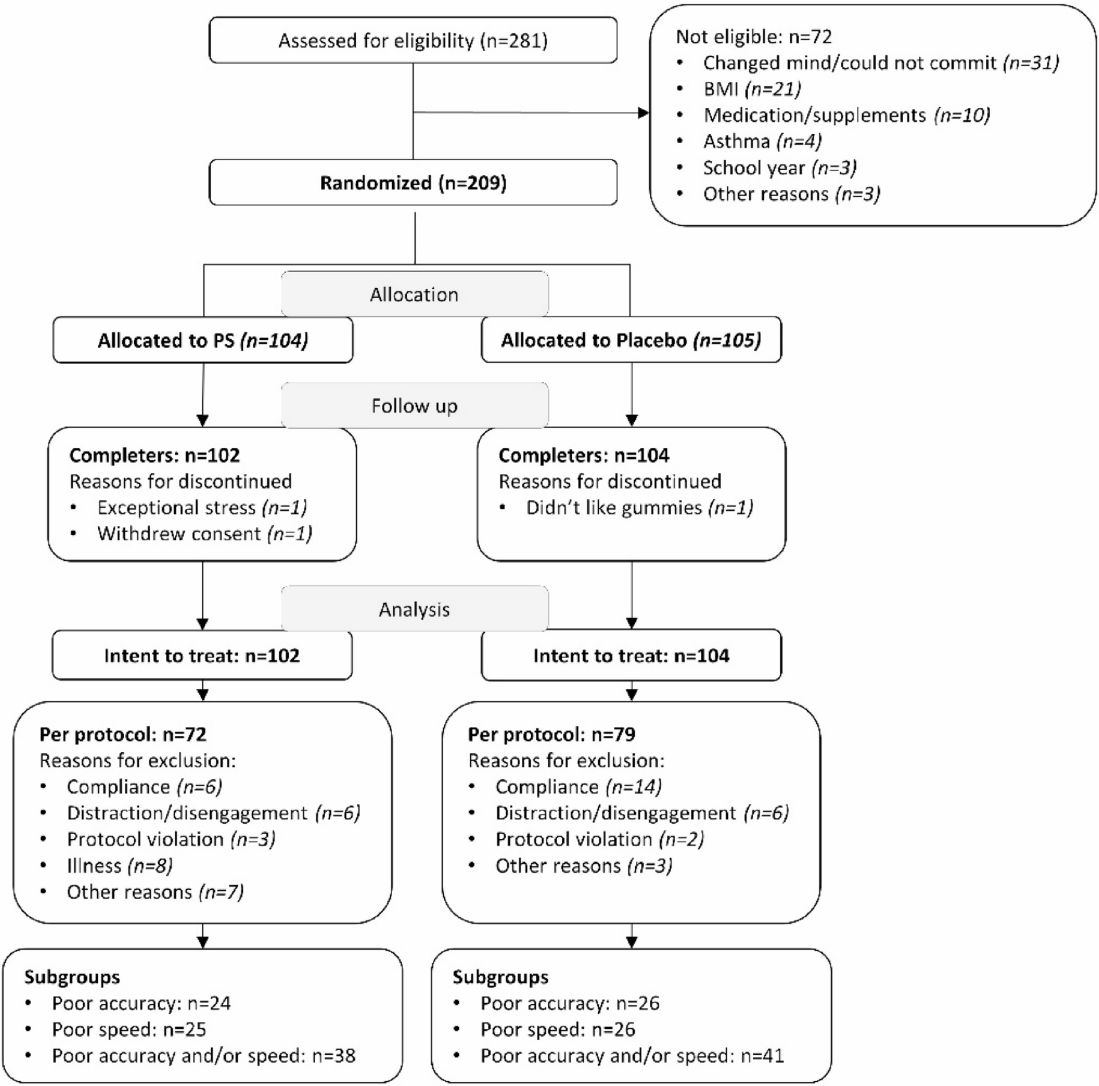
CONSORT flow diagram of participant disposition

Between April 2021 and May 2022, a total of 281 children were screened, and 209 eligible participants were enrolled and randomly assigned to receive sunflower-derived PS (“PS”) or placebo (Fig. 3). As detailed in Table 1, baseline characteristics were similar between the groups.

**Table 1:** Descriptive statistics of main demographic and baseline characteristics in the PP population

Characteristic	PS (*N* = 72)	Placebo (*N* = 79)
Age, yrs. (SD)	10.52 (1.13)	10.67 (1.07)
School year, n (%)		
Year 4	14 (19.44)	16 (20.25)
Year 5	26 (36.11)	22 (27.85)
Year 6	17 (23.61)	17 (21.52)
Year 7	15 (20.83)	24 (30.38)
Sex, n (%)		
Female	40 (55.56)	45 (56.96)
Body mass index, kg/m^2^ (SD)	17.77 (2.22)	17.71 (2.29)
Parent education, n (%)		
Basic	6 (8.33)	6 (7.59)
Secondary	15 (20.83)	19 (24.05)
Bachelor	29 (40.28)	33 (41.77)
Master	21 (29.17)	17 (21.52)
Doctorate	1 (1.39)	4 (5.06)
Eligibility for free school meals, n (%)		
Yes	10 (13.89)	11 (13.92)

Data are presented as mean (SD) unless otherwise stated.

The intent to treat (ITT) population comprised 206 participants (102 PS and 104 placebo). In the ITT population, 26.7% (30 PS and 25 placebo) were classified as having major protocol deviations. The most frequent reason was lack of compliance data (9.7%), which largely reflected incomplete parental reporting or failure to return bottles; overall adherence was high (> 90%). Additional reasons included illness (7.8%) and distraction or disengagement during the COMPASS assessment (5.8%). Overall, 151 subjects (72 PS and 79 placebo) completed the trial without major protocol deviations, thus forming the PP population, and were included in the final analyses. However, not all subjects were able to complete all tasks, resulting in data for some tasks being missing for some participants.

### Primary outcomes

The primary outcomes were defined as composite COMPASS and RAVLT scores at week 12 after initiation of PS or placebo. After adjusting for multiplicity testing, our analysis of the primary outcomes revealed that none of the measured effects differed significantly between study groups (Tables 2 and 3).

**Table 2:** COMPASS composite scores at each visit. Visit 1 (V1) data are Raw means. Post dose data are estimated marginal means derived from the linear mixed model that are adjusted for baseline score

			*Assessment 1*			*Assessment 3*			Model analysis			
	Visit	Group	*N*	Mean	SEM	*N*	Mean	SEM	Analysis	F	*p*	Adj-*p*
Speed of performance	V1	Placebo	79	−0.05	0.05	77	−0.09	0.06	Tr	1.28	0.260	0.83
		PS	71	−0.03	0.06	71	−0.02	0.06				
	V2	Placebo	78	−0.11	0.05	77	−0.04	0.07				
		PS	72	−0.12	0.06	66	0.05	0.09				
	V3	Placebo	78	−0.11	0.06	77	−0.03	0.07				
		PS	70	−0.1	0.06	66	0.13	0.1				
Accuracy of performance	V1	Placebo	79	0.11	0.04	78	−0.11	0.06	Tr	0.48	0.491	0.83
		PS	71	0.14	0.05	71	−0.1	0.06				
	V2	Placebo	79	0.18	0.06	79	−0.13	0.06				
		PS	72	0.23	0.05	70	−0.12	0.06				
	V3	Placebo	79	0.15	0.06	79	−0.13	0.05				
		PS	72	0.22	0.06	67	−0.06	0.05				
Speed of attention	V1	Placebo	79	−0.2	0.05	79	−0.05	0.07	Tr	1.86	0.175	0.74
		PS	70	−0.16	0.06	69	−0.05	0.07				
	V2	Placebo	77	−0.21	0.05	79	−0.01	0.07				
		PS	71	−0.16	0.06	69	0.18	0.11				
	V3	Placebo	79	−0.16	0.06	78	0.05	0.08				
		PS	70	−0.12	0.06	69	0.29	0.12				
Accuracy of attention	V1	Placebo	79	0.06	0.07	79	−0.02	0.08	Tr	0.48	0.488	0.83
		PS	70	0.13	0.05	69	0.02	0.07				
	V2	Placebo	77	0.05	0.08	79	−0.04	0.08				
		PS	71	0.01	0.06	69	−0.09	0.06				
	V3	Placebo	79	0	0.07	78	0.05	0.06				
		PS	70	−0.02	0.07	69	0.05	0.05				
Speed of memory	V1	Placebo	78	0.17	0.08	75	−0.1	0.08	Tr	0.05	0.823	0.94
		PS	72	0.19	0.08	71	0	0.08				
	V2	Placebo	79	0.02	0.07	76	−0.09	0.07				
		PS	72	−0.04	0.08	67	−0.08	0.09				
	V3	Placebo	77	0	0.08	74	−0.14	0.07				
		PS	70	−0.07	0.07	67	−0.05	0.1				
Accuracy of working memory	V1	Placebo	78	0.08	0.07	74	−0.1	0.07	Tr	1.47	0.228	0.80
		PS	72	0.15	0.07	71	−0.06	0.07				
	V2	Placebo	79	0.11	0.07	76	−0.18	0.09				
		PS	72	0.24	0.06	69	−0.09	0.08				
	V3	Placebo	79	−0.03	0.08	73	−0.07	0.08				
		PS	69	0.23	0.07	65	−0.03	0.08				
Accuracy of episodic memory	V1	Placebo	79	0.25	0.06	79	−0.21	0.08	Tr	0.95	0.333	0.83
		PS	71	0.11	0.08	72	−0.27	0.08				
	V2	Placebo	79	0.49	0.06	79	−0.21	0.08				
		PS	72	0.44	0.07	69	−0.15	0.08				
	V3	Placebo	79	0.4	0.08	79	−0.28	0.08				
		PS	72	0.41	0.09	72	−0.25	0.09				

Means (plus SEM) derived from the linear mixed model analysis of composite scores of COMPASS task outcomes measured during two assessments at each study visit for the PP population. V1 baseline comparison p values are presented, along with the model analysis and the Benjamini-Hochberg (B-H) adjusted p value. Tr = main effect of supplementation

**Table 3:** cLLT and RAVLT scores at each visit. Visit 1 (V1) data are Raw means. Post dose data are estimated marginal means derived from the linear mixed model that are adjusted for baseline score

						Model analysis			
	Visit	Group	N	Mean	SEM	Analysis	F	p	Adj-p
cLLT (learning index)	V1	Placebo	78	0.78	0.02	Tr	0.30	0.582	0.830
		PS	72	0.73	0.03				
	V2	Placebo	79	0.84	0.02				
		PS	72	0.83	0.03				
	V3	Placebo	79	0.82	0.03				
		PS	72	0.83	0.02				
cLLT (total displacement)	V1	Placebo	78	21.03	1.82	Tr	2.46	0.119	0.680
		PS	72	23.38	2.30				
	V2	Placebo	79	18.85	2.25				
		PS	72	15.44	1.55				
	V3	Placebo	79	18.71	1.81				
		PS	72	17.60	1.75				
cLLT (delayed displacement)	V1	Placebo	78	−0.21	0.17	Tr	2.57	0.111	0.680
		PS	72	−1.72	0.96				
	V2	Placebo	79	−0.13	0.18				
		PS	72	0.03	0.16				
	V3	Placebo	79	0.56	0.44				
		PS	72	−0.43	0.21				
RAVLT (total learning)	V1	Placebo	79	42.95	1.02	Tr	0.33	0.567	0.830
		PS	70	44.69	0.91				
	V2	Placebo	78	45.23	0.97				
		PS	71	45.75	1.08				
	V3	Placebo	79	44.38	1.01				
		PS	71	46.59	1.06				
RAVLT (immediate recall)	V1	Placebo	79	9.44	0.28	Tr	0.02	0.877	0.940
		PS	70	9.84	0.32				
	V2	Placebo	78	10.23	0.28				
		PS	71	10.11	0.31				
	V3	Placebo	79	9.91	0.28				
		PS	71	10.28	0.31				
RAVLT (delayed recall)	V1	Placebo	79	8.97	0.31	Tr	0.34	0.562	0.830
		PS	70	9.69	0.31				
	V2	Placebo	78	9.46	0.30				
		PS	71	9.34	0.33				
	V3	Placebo	79	9.00	0.31				
		PS	71	9.30	0.33				
RAVLT (delayed recognition)	V1	Placebo	79	13.23	0.22	Tr	0.07	0.797	0.940
		PS	70	13.53	0.21				
	V2	Placebo	78	13.26	0.19				
		PS	71	13.17	0.23				
	V3	Placebo	79	12.58	0.24				
		PS	71	12.76	0.26				

Means (plus SEM) derived from the linear mixed model analysis of computerised location learning task (cLLT) and RAVLT task task outcomes measured during two assessments at each study visit for the PP population. V1 baseline comparison p values are presented, along with the model analysis and the Benjamini-Hochberg (B-H) adjusted p value. Tr = main effect of supplementation

### Secondary outcomes

The secondary endpoints were set as changes in individual scores for the COMPASS tasks at weeks 6 and 12, cLLT and RAVLT at week 6, CSHQ total score at weeks 6 and 12, SSR total score at weeks 6 and 12, actigraph sleep monitoring at week 12, and parent and children VAS scores at weeks 6 and 12 (Supplementary Tables 4–8).

The only statistically significant difference between study groups was observed with the RVIP false alarms score. Children in the placebo group made 1.13 fewer mistakes than the PS group in the false alarms measure of the RVIP test (*p* = 0.022). However, interpretation is substantially limited by the fact that only 28% of the PP population were able to perform this test successfully. No other secondary outcomes showed any statistical difference between the supplement and placebo groups.

### Subgroup analysis of underperformers

Based on previous findings [32], it was hypothesized that the efficacy of supplementation may differentiate across certain subsections of the larger cohort; therefore, analyses were undertaken on three subgroups of children (Supplementary Fig. 1) who consistently performed below the median on accuracy related, speed related, or a combined subgroup of both. Other participants from the PP population who were not included in the subgroup of underperformers were classified as typical performers. The baseline characteristics of the underperforming group were similar between the PS and placebo arms.

The three identified underperforming subgroups showed a consistent statistically significant difference in the total displacement score on the cLLT task (sum of placement errors across five trials; lower scores indicate better performance) which was one of the primary outcome measures. The cLLT task in the current study comprised 5 consecutive attempts (trials) to place simple line drawing images into correct, previously shown positions on an empty grid [23].

**Fig. 4:**
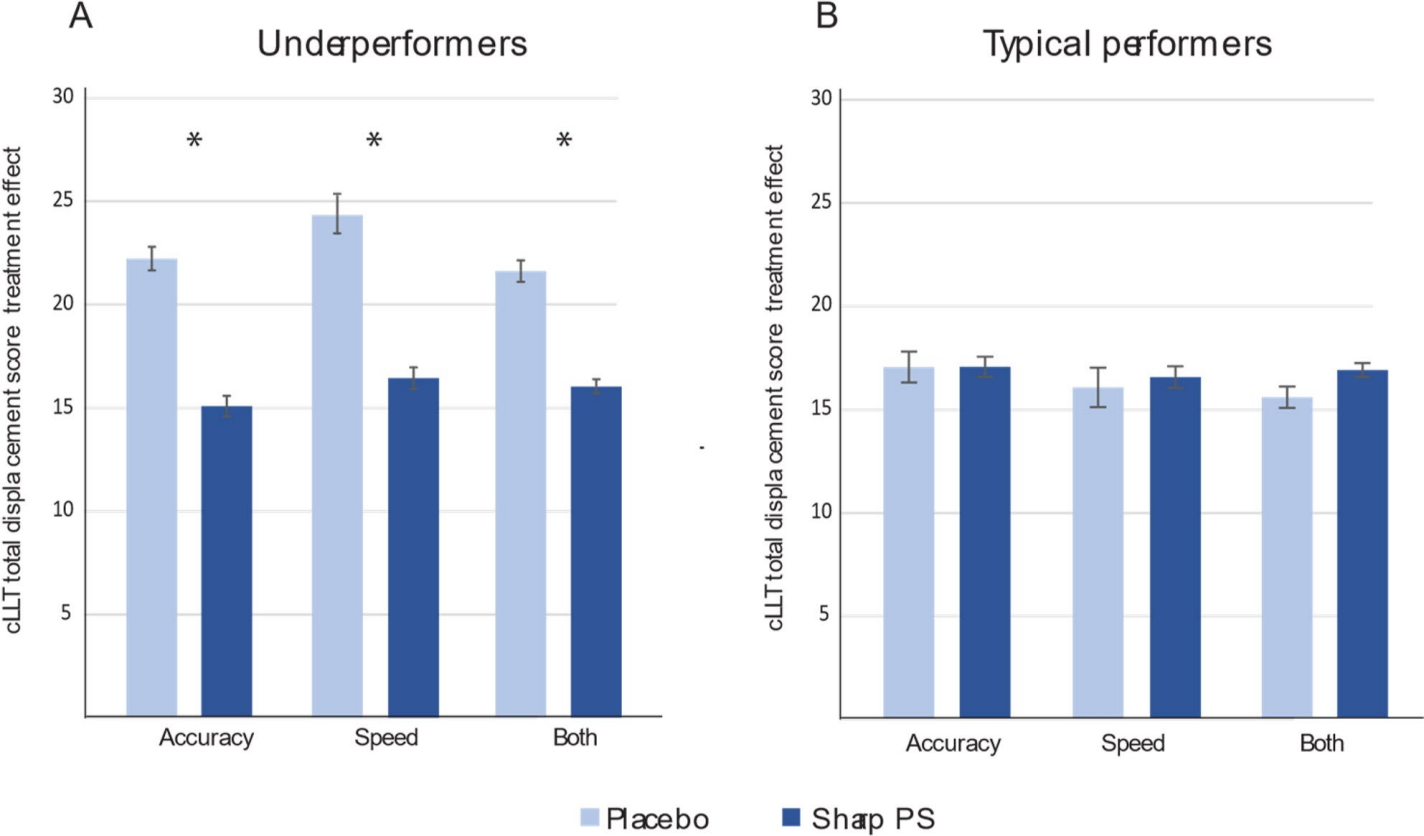
Effect of supplementation on cLLT total displacement scores in subgroups identified as: **A**- underperformers and **B**- typical performers, grouped by composite scores for accuracy, speed, or combined. Mean values are shown. The whiskers denote SEMs. *p < 0.05, compared with placebo.

At baseline, the total displacement score was 24.05 and 19.95 for PS and placebo arms, respectively, in the combined underperformers subgroup. This value decreased to 16.87 in the PS group compared with 21.78 in the placebo group, indicating a greater reduction in errors due to the PS supplementation (*p* < 0.05, Fig. 3A). The total displacement score for PS-supplemented typical children was comparable (16.82) (Fig. 4B). Similarly, significant decline was observed in the accuracy underperformers group, and speed underperformers group (*p* < 0.05, Fig. 3A) with comparable outcomes in the typical performers groups (Fig. 4B).

**Fig. 5:**
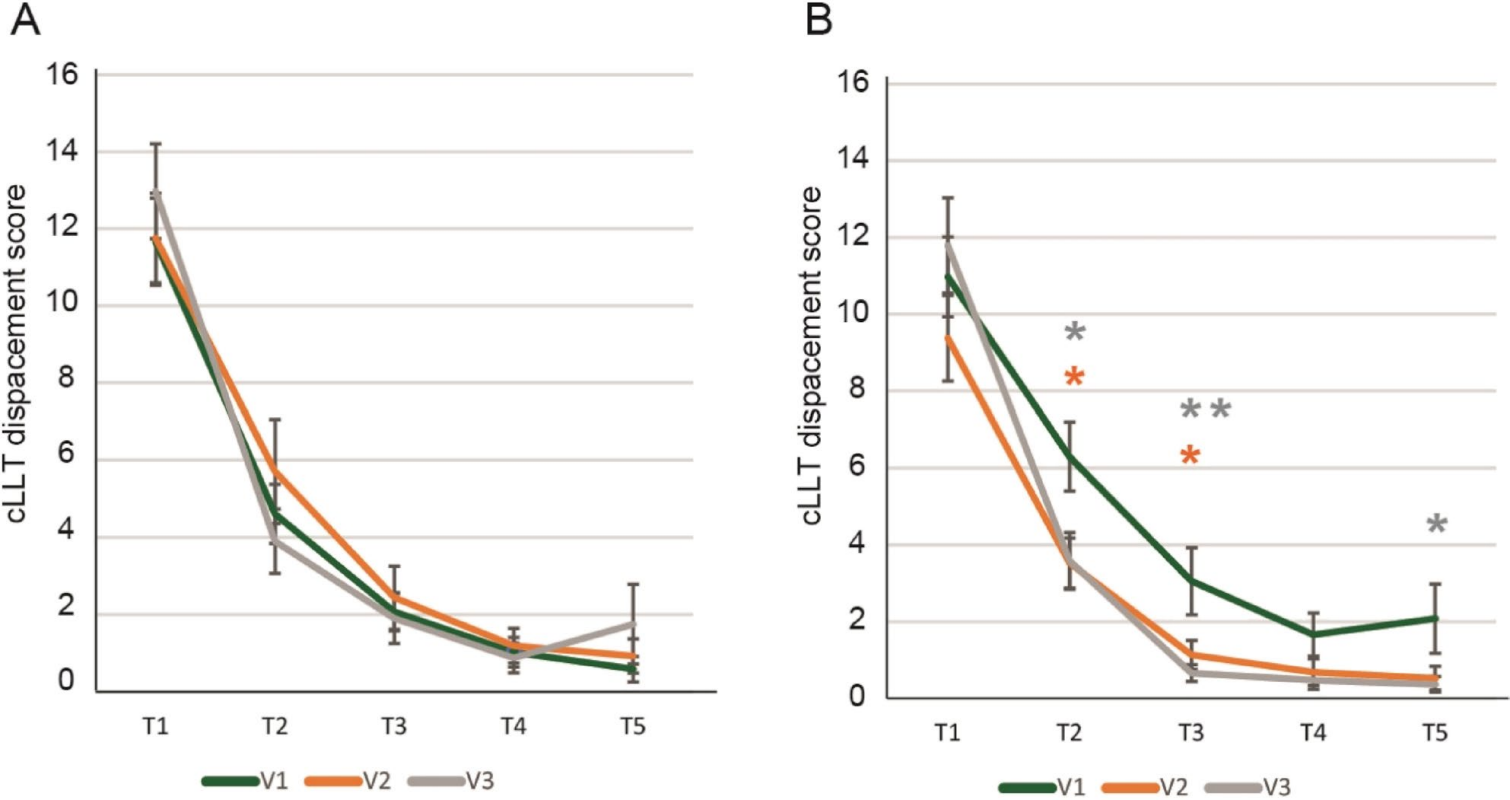
cLLT total displacement scores for PS and placebo groups. Data are unadjusted mean values for the underperforming subgroup receiving (**A**) placebo and (**B**) PS (combined subgroup). T1–5: CLLT displacement attempts 1–5;. *p < 0.05; **p < 0.01. Asterisk’s color indicates a comparison with V1

To demonstrate differences in the speed of learning, displacement scores for each of the attempts (trials 1–5) were analyzed for the two supplementation groups across all three visits (V1-V3) (Fig. 5). There were no statistically significant differences in the speed of learning between the study groups. When comparing the differences within the groups, in contrast to the placebo group (Fig. 5A), where there were no significant changes in cLLT displacement scores throughout the trials in the PS-supplemented participants (Fig. 5B) made fewer errors in trials 2 (*p* = 0.018) and 3 (*p* = 0.019) at visit 2 and trials 2 (*p* = 0.026), 3 (*p* = 0.003), and 5 (*p* = 0.020) at visit 3, compared with baseline (V1).

### Safety profile

An equal number of participants in the PS and placebo groups experienced at least 1 adverse event (AE) (62 vs. 65, respectively). The most frequently reported AEs in both groups were aches and pains (39 in PS recipients vs. 28 in the placebo group). Most AEs were mild (50% in the PS group and 55.4% in the placebo group) or moderate (24.2% and 26.2%, respectively) in severity. Severe AEs were reported in 11.3% of the PS group and 7.7% of those who received placebo. No serious AEs were reported, and no participants discontinued supplementation due to AEs. Two AEs, both gastrointestinal, were considered possibly related to the study intervention, but both AEs were mild in severity.

## Discussion

The rationale for this randomized, double-blind, placebo-controlled clinical trial was the lack of information regarding PS enhancement effect in healthy children. Further, the cognitive benefits demonstrated in other studies with soybean PS supplementation led us to assume there would be a similar effect due to the similarity between soybean and sunflower PS. Despite our hypothesis, our study failed to demonstrate such an effect in healthy children aged 8 to 12 years. No statistically significant differences were observed between the PS and placebo groups for any of the primary or secondary outcome measures. PS, however, increased visuospatial learning in an underperforming subgroup of children and did not elicit any significant safety concerns. The primary and secondary findings suggest that our PS supplementation regimen and study design may be insufficient to demonstrate cognitive improvements in this cohort of healthy school-aged children with no cognitive complaints. A potential explanation for this lack of efficacy is that the development of general cognitive function has been shown to reach its upper limit by early adolescence. Consequently, it is likely that a significant proportion of participants had a limited capacity for further cognitive enhancement through PS supplementation [33]. Thus, when designing the trial, we postulated that children performing below median at baseline in cognitive performance measures (underperformers) would be more likely to show improvements following PS supplementation, as they would have greater potential for cognitive advancement.

In this study, the underperformers were classified as children who scored below the median on the 7 COMPASS composite scores at baseline. We showed that those who consumed PS experienced significantly larger improvements in cLLT displacement scores throughout the study than those who were given placebo. This finding suggests greater improvements in visuospatial learning in PS-supplemented underperforming participants, which were observed throughout the study period. Although the results should be considered tentatively due to an increased risk of type 1 error, the improvement in cLLT displacement score was demonstrated in all three underperforming subgroups and thus should be regarded as valid evidence of improvement.

The improvement in visuospatial learning in underperformers, as measured by the cLLT, is a promising finding that merits further investigation. The mechanisms by which PS may improve visuospatial learning in children are unknown, although potential mechanisms center on its ability to support cholinergic activity and provide neuroprotection to the hippocampus, based on animal and human studies. In rats that were exposed to a neurotoxin, Ye and colleagues found that soybean-derived PS restored memory function in the Morris water maze task, an effect that was accompanied by mitigation of damage to hippocampal cholinergic cells [34]. Similar results have been reported for krill-derived PS in aged rats [35]. The hippocampus is important for spatial navigation, learning, and memory [36], and deficits in spatial memory can occur after hippocampal damage [37].

A relationship between visuospatial memory and cholinergic neurotransmitters has also been identified. For example, Winkler et al. [38] linked neurotransmitters to learning and memory by grafting acetylcholine-producing cells into rats with impaired cholinergic innervation of the neocortex—a structure that cooperates with the hippocampus to influence learning and memory [39]. By increasing neocortical acetylcholine levels, the authors observed that grafted rats improved significantly in a spatial navigation task, demonstrating that the cholinergic system is essential for spatial memory. Similarly, in a human study, administration of a cholinergic antagonist significantly deteriorated visuospatial processing and memory [40]. Thus, it is possible that sunflower-derived PS enhances cholinergic activity by upregulating acetylcholine levels or rejuvenating hippocampal cholinergic cells to improve visuospatial learning in underperforming children.

Several characteristics of the current study design and conduct may have impacted the findings. First, some children struggled with the length of the study visits and task repetition. Each visit lasted over 3 h, wherein most tasks were performed 2 or 3 times in each visit. This design has been previously applied and has yielded successful results in adult populations. The current study resulted in significant variability in participants’ performance. Also, despite initial training by the research team, some tasks appeared inappropriate for this cohort of children, as many had difficulties completing the tasks per the instructions. Future trials should adopt more age-appropriate designs, employing simplified and shorter protocols with straightforward tasks that allows cognitive changes to be detected..

Additionally, this study might have been subject to certain technical constraints. The low adherence to the study procedures may have impacted the robustness of the findings. Over 25% of randomized participants were excluded from the PP analysis, resulting in a smaller than planned sample size. Further, a dose of 100 mg PS per day has been previously used in studies with adults [21, 41], but it is possible that higher doses are required to achieve cognitive-enhancing effects in school-aged children. In studies of children with ADHD, improvements in memory and attentional symptoms have been observed with a higher dose (200 mg daily) [42–44]. Finally, differences in fatty acid composition between sunflower-derived PS and other sources were not tested previously, and although such differences were not anticipated to influence the outcomes, they may be worthwhile to explore in future studies.

In summary, 12 weeks of supplementation with sunflower-derived PS was not associated with significant changes in cognitive performance, sleep, or mood in healthy children aged 8 to 12 years. However, improvements in visuospatial learning were detected in a subset of children who were defined as underperformers. These findings merit further examination, including a more detailed analysis of underperformers, using age-appropriate cognitive assessments in children who experience some deficits in cognitive performance to support the robustness of these conclusions and to confirm the potential benefits of Sharp PS^®^ Green. Supplementation with sunflower-derived PS was well tolerated and appears to be safe in healthy school-aged children.

## Supplementary Information

Supplementary Material 1: Table S1: Tasks completed. Table S2: Child mood VAS. Table S3: Baseline characteristics of underperformers. Table S4: Means (plus SEM) derived from the linear mixed model analysis of COMPASS task outcomes measured during three assessments at each study visit for PP population. Table S5: Means (plus SEM) derived from the linear mixed model analysis of COMPASS task outcomes measured during two assessments at each study visit for PP population. Table S6: Means (plus SEM) derived from the linear mixed model analysis of mood visual analogue scales measured during two assessments at each study visit for PP population. Table S7: Means (plus SEM) derived from the linear mixed model analysis of parent visual analogue scales for PP population. Table S8: Means (plus SEM) derived from the linear mixed model analysis of sleep actigraphy for PP population.

## Data Availability

The datasets generated and/or analysed during the current study are proprietary data and are not publicly available but are available from the corresponding author on reasonable reques.
