# Supplementary material for: The cognitive effects of supplementation with sunflower phosphatidyl serine in healthy children aged 8 to 12 years: a randomized controlled trial
Source: Nutr J. 2025 Nov 29;25:3. doi: 10.1186/s12937-025-01264-9 (PMC12772107; doi:10.1186/s12937-025-01264-9)
Supplement: Supplementary file 1 — Supplementary Material 1: Table S1: Tasks completed. Table S2: Child mood VAS. Table S3: Baseline characteristics of underperformers. Table S4: Means (plus SEM) derived from the linear mixed model analysis of COMPASS task outcomes measured during three assessments at each study visit for PP population. Table S5: Means (plus SEM) derived from the linear mixed model analysis of COMPASS task outcomes measured during two assessments at each study visit for PP population. Table S6: Means (plus SEM) derived from the linear mixed model analysis of mood visual analogue scales measured during two assessments at each study visit for PP population. Table S7: Means (plus SEM) derived from the linear mixed model analysis of parent visual analogue scales for PP population. Table S8: Means (plus SEM) derived from the linear mixed model analysis of sleep actigraphy for PP population. [file 12937_2025_1264_MOESM1_ESM.docx]

**The cognitive effects of supplementation with Sharp PS® Green in healthy children aged 8 to 12 years: A randomized controlled trial**

Marina Friling^1†*^, Philippa A. Jackson^2†^, David Kennedy^2^, Fiona Dodd^2^, Ellen Smith^2^, Arava Lavie^1^, Adrian Lopresti^3^, Eran Ivanir^1‡^, Jonna Jalanka^4‡^

^1^IFF Health, Migdal Haemek, Israel, ^2^Brain, Performance and Nutrition Research Centre, Northumbria University, Newcastle upon Tyne, UK, ^3^Clinical Research Australia, Australia, ^4^IFF Health & Biosciences, Kantvik, Finland

*CORRESPONDENCE

Marina Friling; marina.friling@iff.com

†These authors have contributed equally to this work and share first authorship

‡These authors have contributed equally to this work and share last authorship

Supplementary Material

# All inclusion and exclusion criteria and protocol deviations

INCLUSION CRITERIA

- Participants in good health, as reported by themselves and their parent/guardian.
- Healthy children aged 8 to 12 years and enrolled in school years 4 to 7 at the time of giving consent
- Have been speaking English at school since reception (kindergarten)
- Willingness of the children and parents to give their written informed consent, according to good clinical practice (GCP) and local regulations, and being able to participate in all scheduled visits, treatment plans, tests, and other trial procedures
- Children with a normal sex-related and age-related BMI according to the local NHS guidelines (3rd to 90th percentiles)

EXCLUSION CRITERIA

Participants were not eligible to take part if the any of the following applied:

- Had relevant allergy or known hypersensitivity to one of the ingredients contained in the investigational product
- Taking any illicit, herbal, or recreational drugs, including alcohol and tobacco.
- Taking any prescribed or over the counter (OTC; available without prescription from the chemist/pharmacist) medication against chronic or non- chronic illnesses.
- Had used dietary supplements within the last 4 weeks
- Were diagnosed with ADHD, dyslexia, or any neurodevelopmental disorder or learning difficulty.
- Suffered from visual (including color blindness) or hearing impairment (that may impact task performance in the opinion of the PI).
- Had any serious illness, cognitive impairment, or medical disorder that may confound with study results or interfere with compliance.
- Had any other active or unstable medical condition that, in the opinion of the PI, may adversely affect the participant's ability to complete the study
- Were experiencing exceptional social/family stressors.
- Consumed more than one portion (>100 g) per week of the following dietary sources high in phosphatidylserine: Oily fish, such as salmon, mackerel, herring, tuna, and eel. Animal internal organs such as liver, kidney, brain, and heart
- Had followed specific diet, e.g., high protein diet, within 30 days prior to study start
- Serious diet change, e.g. Ketogenic or vegan, within 30 days prior to study start.
- Consumption of > 250 mg/day of caffeine.
- Were unable to complete all of the study assessments
- Were currently participating in other clinical or nutrition intervention studies or have in the past 8 weeks
- Were non-compliant with regard to consumption of supplement

# Table S1. Tasks completed.

| Task | Description | Scoring | Domain |
| --- | --- | --- | --- |
| RAVLT | A list of words is presented several times, after which distractor words are presented. Participant is asked to indicate for each word whether it was in the original list. |  |  |
| Total learning |  | Sum of words recalled across the 5 trials |  |
| Immediate recall |  | Number of words recalled after the distractor list – first trial following the 5 learning rounds | Episodic memory |
| Delayed recall |  | Number of words recalled after the break | Episodic memory |
| Delayed recognition |  | Number of words recognized after the break | Episodic memory |
| COMPASS | A bespoke collection of tasks with fully randomized parallel versions of each task delivered at each assessment for each individual. |  |  |
| Immediate word recall | A unique set of 12 words is presented to the participant once every 3 seconds, with a stimulus duration of 1 second. Participants are allowed 60 seconds to write down as many of the words as possible. | Accuracy (% correct) | Episodic memory |
| Simple reaction time | An upwards pointing arrow is presented at irregular intervals between 1 and 3 seconds (total 30 stimuli); participants respond as quickly as they can as soon as they see the arrow appear. | Reaction time (msec) | Attention |
| Choice reaction time | Participants indicate the direction of a left or right pointing arrow. A total of 30 stimuli (arrows) are presented at irregular intervals between 1 and 3 seconds. | Accuracy (% correct) and reaction time for correct responses (msec) | Attention |
| Four-choice react time | Arrows appear in a random order at irregular intervals between 1 and 3 seconds. The participant is required to indicate the direction of the arrow as quickly as possible (total 28 stimuli). | Accuracy (% correct) and reaction time for correct responses (msec) | Attention |
| Arrows flankers | Five symbols appear at irregular intervals between 1 and 3 seconds, with the center symbol always being an arrow pointing to the left or right. The participant indicates the direction of the central arrow. The flanking pairs of symbols are congruent arrows, incongruent arrows, or squares. Each stimulus remains on screen until the response is registered (total 81 stimuli). | Accuracy (% correct) and reaction time for correct responses (msec) for each of the three types of stimuli and overall | Attention |
| Digit vigilance | A fixed number appears on the right, and a series of changing numbers appears on the left at 150 per minute (total 15 target stimuli). Participants respond when the number on the left matches the number on the right. | Accuracy (% correct), reaction time for correct responses (msec), and number of false alarms (hitting the button when mismatch occurs) | Attention |
| Rapid Visual Information Processing (RVIP) | Participant monitors a continuous series of single digits for targets of 3 consecutive odd or even numbers. The digits appear at 100 per minute for 3 minutes, with 8 correct target strings in each minute presented in pseudo-random order. The participant responds to the detection of a target string. | Accuracy (% detected), false alarms (hitting the button when mismatch occurs), and reaction time for correct responses (msec) | Attention |
| Stroop | A series of 40 colors (RED, YELLOW, GREEN, BLUE) are presented one at a time in colored font. Participants respond to the color in which the word is written and not the color the word depicts. | Accuracy (% correct), overall reaction time for correct responses (msec), and the same individual outcomes for congruent stimuli and incongruent stimuli | Attention |
| Corsi blocks task | Nine identical blue squares appear in non-overlapping random positions. A set number of blocks change color from blue to red in a randomly generated sequence. Subjects recall the sequence and repeat it by clicking on the blocks. The task is repeated 5 times at each level of difficulty, starting with 5 blocks, until the participant can no longer correctly recall the sequence. | Span score (averaging the level of the last three correctly completed trials) | Spatial working memory |
| Numeric working memory | Five random digits from 1–9 are presented sequentially for the participant to hold in memory, followed by a series of 30 probe digits (15 targets and 15 distractors), for each of which the participant indicates whether it was in the original series (3 separate trials). | Accuracy (% correct) and mean reaction time for correct responses (msec) | Working memory |
| 2-back | 45 letters in uppercase and lowercase are shown one at a time for 500 msec. The participant decides if the currently displayed letter (ignoring case) is the same as the letter shown 2 letters previously. | Accuracy (% correct), errors (number of responses in the absence of target stimuli), reaction time for correct responses (msec) | Working memory |
| Delayed word recall | 12 words are presented at 1 every 3 seconds, with a stimulus duration of 1 second. After ~20 minutes, participants write down as many words as possible. | Accuracy (% correct) | Episodic memory |
| Delayed picture recognition | 12 pictures are presented at 1 every 3 seconds, with a stimulus duration of 1 second. After ~20 minutes, participants differentiate between the 12 pictures and 12 decoy pictures. | Accuracy (% correct), and reaction time for correct responses (msec) | Episodic memory |
| Delayed word recognition | 12 words are presented at 1 every 3 seconds, with a stimulus duration of 1 second. After ~20 minutes, participants differentiate between the 12 target words and 12 decoy words. | Accuracy (% correct), and reaction time for correct responses (msec) | Episodic memory |
| Computerized location learning task (cLLT) | **Learning phase**: A 5 x 5 grid containing 10 pictures of objects is presented for 15 seconds. Following a 10 sec delay, an empty grid is shown, and the original 10 pictures are presented on the right side of the screen. Participants are instructed to drag and drop the objects back into the location they had occupied in the grid. The task is repeated five times using the same display of objects during the learning phase with a pause of 5 s between each trial. Task outcomes: Total displacement score, calculated as the sum of the five learning trials (sum of errors made for each object, calculated by counting the number of cells the object had to be moved both horizontally and vertically to be in the correct location); lower scores indicate better performance. Learning index (calculated as the average relative difference in normalized displacement score between trials in which the letters A–E represent the Displacement Scores for the first five trials [((A - B)/A + (B - C)/B + (C - D)/C + (D - E)/D)/4]); scores range from 0-1, with 1 being the highest possible score, indicating better performance.  **Delayed phase**: The delayed trial will take place at least 30 min after completion of the learning phase. Participants are again asked to place the objects in the correct location on the empty grid with no further prompting. | Delayed displacement (calculated as the difference between displacement score on the final learning trial, E, and the delayed trial); scores range from 0 as the highest score, and increasing negative numbers as an index of decay (lower performance) | Spatial memory |

# Table S2. Child mood VAS

| **VAS Question** | **Scale** | **Interpretation** |
| --- | --- | --- |
| How relaxed do you feel right now? | 0=not at all, 100=extremely | Higher score indicates more relaxed state |
| How alert do you feel right now? | 0=not at all, 100=extremely | Higher score indicates more alert state |
| How jittery do you feel right now? | 0=not at all, 100=extremely | Higher score indicates more jittery state |
| How tired do you feel right now? | 0=not at all, 100=extremely | Higher score indicates more tired state |
| How tense do you feel right now? | 0=not at all, 100=extremely | Higher score indicates more tense state |
| On the following screen, use the mouse to click an ‘X’ at a point on the scale which best represents your current level of headache | 0=no headache, 100=extreme headache | Higher score indicates more severe headache |
| How would you rate your overall mood right now? | 0=very bad mood 100=very good mood | Higher score indicates better mood |
| How mentally fatigued do you feel right now? | 0=not at all, 100=extremely | Higher score indicates more mental fatigue |
| How well do you think you’re performing? | 0=really well, 100=not at all well | Lower score indicates better performance |
| Do you normally feel happy/sad? | 0=happy, 100=sad | Lower score indicates happier state |
| Do you normally feel stressed/calm? | 0=stressed, 100=calm | Higher score indicates calmer state |

# Parent VAS on child’s overall performance

How attentive is your child?

How do you feel your child is performing at school?

How well do you think your child is performing schoolwork at home?

How happy is your child?

How stressed is your child?

For the first 4 questions the scale is 0=not very, 100=very, higher score indicates better state. For the stress question, the scale is reversed (0=very and 100=not very), higher score indicates less stressed state.

# Table S3. Baseline characteristics of underperformers

| Characteristic | PS  (n=38) | Placebo  (n=41) | Total  (n-79 | P-value |
| --- | --- | --- | --- | --- |
| Age, yrs (SD) | 10.22 (0.96) | 10.4 (1.1) | 10.31 (1.04) | 0.433^1^ |
| School year, n (%) |  |  |  | 0.331^2^ |
| Year 4 | 8 (21.05) | 11 (26.83) | 19 (24.05) |  |
| Year 5 | 20 (52.63) | 14 (34.15) | 34 (43.04) |  |
| Year 6 | 6 (15.79) | 7 (17.07) | 13 (16.46) |  |
| Year 7 | 4 (10.53) | 9 (21.95) | 13 (16.46) |  |
| Sex, n (%) |  |  |  |  |
| Female | 20 (52.63) | 24 (58.54) | 44 (55.7) | 0.598^2^ |
| Body mass index, kg/m^2^ (SD) | 17.68 (2) | 17.73 (2.45) | 17.7 (2.23) | 0.917^1^ |
|  |  |  |  | 0.679^2^ |
| Basic | 4 (10.53) | 2 (4.88) | 6 (7.59) |  |
| Secondary | 8 (21.05) | 10 (24.39) | 18 (22.78) |  |
| Bachelor | 15 (39.47) | 21 (51.22) | 36 (45.57) |  |
| Master | 10 (26.32) | 7 (17.07) | 17 (21.52) |  |
| Doctorate | 1 (2.63) | 1 (2.44) | 2 (2.53) |  |
| Eligibility for free school meals, n (%) |  |  |  |  |
| Yes | 8 (21.05) | 6 (14.63) | 14 (17.72) | 0.455^2^ |

Data are presented as mean (SD) unless otherwise stated; ^1^p-values are derived from independent t-test; ^2^p-values are derived from chi square test.

# Table S4. Means (plus SEM) derived from the linear mixed model analysis of COMPASS task outcomes measured during three assessments at each study visit for PP population.

|  |  |  |  | Assessment 1 | | | Assessment 2 | | | Assessment 3 | |  |  |  |
| --- | --- | --- | --- | --- | --- | --- | --- | --- | --- | --- | --- | --- | --- | --- |
|  | visit | Arm | N | Mean | SEM | N | Mean | SEM | N | Mean | SEM | Analysis | F | p |
| Simple reaction time (ms) | V1 | Placebo | 79 | 442.26 | 19.85 | 79 | 483.55 | 15.25 | 79 | 502.99 | 17.39 | Tr | 0.39 | 0.54 |
|  |  | PS | 72 | 454.02 | 15.22 | 72 | 509.34 | 20.70 | 72 | 554.76 | 34.34 | Tr x D | 0.03 | 0.85 |
|  | V2 | Placebo | 79 | 433.15 | 13.60 | 79 | 526.23 | 26.64 | 79 | 539.08 | 22.93 |  |  |  |
|  |  | PS | 72 | 441.71 | 13.21 | 72 | 540.44 | 23.22 | 72 | 587.62 | 31.02 |  |  |  |
|  | V3 | Placebo | 78 | 481.72 | 21.47 | 79 | 564.91 | 21.64 | 79 | 600.62 | 33.20 |  |  |  |
|  |  | PS | 72 | 463.21 | 14.40 | 72 | 617.26 | 30.69 | 72 | 626.14 | 32.70 |  |  |  |
| Choice reaction time (%correct) | V1 | Placebo | 79 | 94.94 | 0.52 | 79 | 95.74 | 0.47 | 79 | 95.49 | 0.54 | Tr | 0.01 | 0.92 |
|  |  | PS | 71 | 95.35 | 0.46 | 69 | 96.23 | 0.41 | 70 | 95.38 | 0.54 | Tr x D | 0.24 | 0.62 |
|  | V2 | Placebo | 79 | 94.35 | 0.68 | 79 | 95.99 | 0.51 | 79 | 95.61 | 0.52 |  |  |  |
|  |  | PS | 72 | 94.21 | 0.57 | 72 | 95.46 | 0.49 | 72 | 95.97 | 0.44 |  |  |  |
|  | V3 | Placebo | 79 | 95.40 | 0.53 | 78 | 96.24 | 0.57 | 79 | 96.62 | 0.52 |  |  |  |
|  |  | PS | 72 | 95.83 | 0.50 | 72 | 96.11 | 0.54 | 70 | 96.95 | 0.48 |  |  |  |
| Choice reaction time (ms) | V1 | Placebo | 79 | 634.60 | 19.28 | 79 | 725.14 | 35.46 | 79 | 746.05 | 28.94 | Tr | 2.63 | 0.11 |
|  |  | PS | 71 | 648.59 | 21.31 | 69 | 729.38 | 39.36 | 70 | 759.65 | 40.57 | Tr x D | 0.16 | 0.69 |
|  | V2 | Placebo | 79 | 634.64 | 18.14 | 79 | 720.71 | 24.01 | 79 | 734.39 | 27.43 |  |  |  |
|  |  | PS | 72 | 626.48 | 19.65 | 72 | 782.60 | 40.10 | 72 | 844.60 | 53.76 |  |  |  |
|  | V3 | Placebo | 79 | 667.44 | 22.04 | 78 | 806.56 | 47.10 | 79 | 765.07 | 26.54 |  |  |  |
|  |  | PS | 72 | 689.96 | 25.24 | 72 | 908.50 | 55.57 | 70 | 839.64 | 48.45 |  |  |  |
| 4-choice reaction time (%correct) | V1 | Placebo | 79 | 97.92 | 0.36 | 79 | 97.70 | 0.42 | 79 | 97.11 | 0.46 | Tr | 1.47 | 0.23 |
|  |  | PS | 71 | 98.34 | 0.34 | 72 | 97.37 | 0.71 | 72 | 96.63 | 0.76 | Tr x D | 2.14 | 0.15 |
|  | V2 | Placebo | 79 | 98.10 | 0.35 | 79 | 96.93 | 0.49 | 79 | 97.06 | 0.45 |  |  |  |
|  |  | PS | 72 | 98.36 | 0.26 | 72 | 98.02 | 0.52 | 72 | 98.07 | 0.39 |  |  |  |
|  | V3 | Placebo | 79 | 97.42 | 0.44 | 79 | 97.29 | 0.50 | 79 | 97.33 | 0.50 |  |  |  |
|  |  | PS | 72 | 97.77 | 0.43 | 71 | 97.03 | 0.48 | 72 | 97.62 | 0.39 |  |  |  |
| 4-choice reaction time (ms) | V1 | Placebo | 79 | 829.71 | 25.59 | 79 | 889.82 | 47.76 | 79 | 865.69 | 29.44 | Tr | 1.11 | 0.30 |
|  |  | PS | 71 | 845.43 | 27.02 | 72 | 977.37 | 52.24 | 72 | 889.32 | 33.56 | Tr x D | 2.92 | 0.09 |
|  | V2 | Placebo | 79 | 828.89 | 29.25 | 79 | 912.53 | 42.23 | 79 | 875.45 | 27.72 |  |  |  |
|  |  | PS | 72 | 861.67 | 34.65 | 72 | 1024.77 | 59.87 | 72 | 1008.69 | 63.09 |  |  |  |
|  | V3 | Placebo | 79 | 841.86 | 28.38 | 79 | 1019.81 | 49.33 | 79 | 904.43 | 29.64 |  |  |  |
|  |  | PS | 72 | 868.60 | 34.14 | 71 | 993.52 | 47.20 | 72 | 993.79 | 50.26 |  |  |  |
| Arrow flankers (neutral - %correct) | V1 | Placebo | 77 | 98.81 | 0.25 | 77 | 98.43 | 0.28 | 76 | 97.75 | 0.39 | Tr | 1.02 | 0.32 |
|  |  | PS | 65 | 98.78 | 0.29 | 65 | 98.52 | 0.30 | 68 | 98.47 | 0.33 | Tr x D | 1.58 | 0.21 |
|  | V2 | Placebo | 76 | 98.63 | 0.29 | 77 | 97.40 | 0.51 | 78 | 97.60 | 0.54 |  |  |  |
|  |  | PS | 67 | 98.94 | 0.24 | 68 | 98.04 | 0.34 | 69 | 98.37 | 0.32 |  |  |  |
|  | V3 | Placebo | 78 | 97.81 | 0.41 | 76 | 98.03 | 0.42 | 76 | 98.46 | 0.32 |  |  |  |
|  |  | PS | 69 | 98.61 | 0.28 | 69 | 97.89 | 0.35 | 67 | 97.82 | 0.50 |  |  |  |
| Arrow flankers (neutral RT - ms) | V1 | Placebo | 77 | 810.15 | 35.35 | 77 | 945.52 | 67.49 | 76 | 872.11 | 43.51 | Tr | 1.52 | 0.22 |
|  |  | PS | 65 | 808.50 | 34.08 | 65 | 836.13 | 30.60 | 68 | 880.60 | 45.29 | Tr x D | 0.19 | 0.66 |
|  | V2 | Placebo | 76 | 799.24 | 29.98 | 77 | 955.42 | 52.57 | 78 | 953.06 | 52.31 |  |  |  |
|  |  | PS | 67 | 843.56 | 46.70 | 68 | 1030.80 | 76.84 | 69 | 1051.39 | 101.49 |  |  |  |
|  | V3 | Placebo | 78 | 834.51 | 36.14 | 76 | 995.41 | 42.27 | 76 | 916.88 | 33.66 |  |  |  |
|  |  | PS | 69 | 833.12 | 33.20 | 69 | 1122.22 | 71.57 | 67 | 1054.01 | 91.82 |  |  |  |
| Arrow flankers (congruent - %correct) | V1 | Placebo | 77 | 99.19 | 0.26 | 77 | 97.83 | 0.48 | 76 | 98.68 | 0.29 | Tr | 1.32 | 0.25 |
|  |  | PS | 65 | 98.72 | 0.27 | 65 | 98.59 | 0.31 | 68 | 98.65 | 0.31 | Tr x D | 0.04 | 0.85 |
|  | V2 | Placebo | 76 | 98.79 | 0.31 | 77 | 97.89 | 0.43 | 78 | 98.61 | 0.29 |  |  |  |
|  |  | PS | 67 | 98.82 | 0.32 | 68 | 98.65 | 0.31 | 69 | 98.67 | 0.33 |  |  |  |
|  | V3 | Placebo | 78 | 99.14 | 0.23 | 76 | 98.03 | 0.43 | 76 | 98.08 | 0.43 |  |  |  |
|  |  | PS | 69 | 99.09 | 0.27 | 69 | 98.85 | 0.32 | 67 | 98.32 | 0.45 |  |  |  |
| Arrow flankers (congruent RT - ms) | V1 | Placebo | 77 | 802.38 | 28.03 | 77 | 988.14 | 62.43 | 76 | 896.86 | 40.63 | Tr | 0.83 | 0.36 |
|  |  | PS | 65 | 826.24 | 37.61 | 65 | 874.75 | 40.67 | 68 | 896.04 | 43.29 | Tr x D | 0.01 | 0.92 |
|  | V2 | Placebo | 76 | 808.65 | 26.70 | 77 | 982.27 | 59.46 | 78 | 913.16 | 40.48 |  |  |  |
|  |  | PS | 67 | 868.47 | 46.38 | 68 | 1041.56 | 58.49 | 69 | 990.68 | 55.24 |  |  |  |
|  | V3 | Placebo | 78 | 874.52 | 35.34 | 76 | 1085.14 | 66.55 | 76 | 1004.41 | 51.59 |  |  |  |
|  |  | PS | 69 | 893.55 | 42.52 | 69 | 1188.22 | 91.85 | 67 | 1099.24 | 76.61 |  |  |  |
| Arrow flankers (incongruent - %correct) | V1 | Placebo | 77 | 88.80 | 2.27 | 77 | 90.96 | 1.55 | 76 | 87.45 | 2.16 | Tr | 3.11 | 0.08 |
|  |  | PS | 65 | 93.40 | 1.57 | 65 | 90.13 | 1.54 | 68 | 87.01 | 2.62 | Tr x D | 2.26 | 0.13 |
|  | V2 | Placebo | 76 | 91.39 | 1.13 | 77 | 89.56 | 1.59 | 78 | 90.60 | 1.75 |  |  |  |
|  |  | PS | 67 | 90.36 | 1.94 | 68 | 86.89 | 2.37 | 69 | 85.63 | 2.66 |  |  |  |
|  | V3 | Placebo | 78 | 90.54 | 1.82 | 76 | 91.17 | 1.69 | 76 | 90.57 | 1.80 |  |  |  |
|  |  | PS | 69 | 86.17 | 2.64 | 69 | 88.16 | 2.38 | 67 | 88.87 | 1.80 |  |  |  |
| Arrow flankers (incongruent RT - ms) | V1 | Placebo | 77 | 930.16 | 34.76 | 77 | 1099.84 | 64.93 | 76 | 1034.41 | 55.73 | Tr | 2.13 | 0.15 |
|  |  | PS | 65 | 954.10 | 41.49 | 65 | 1000.35 | 45.48 | 68 | 1022.95 | 49.95 | Tr x D | 2.06 | 0.15 |
|  | V2 | Placebo | 76 | 913.96 | 31.26 | 77 | 1075.18 | 44.21 | 78 | 1024.83 | 38.92 |  |  |  |
|  |  | PS | 67 | 1045.14 | 89.71 | 68 | 1186.01 | 63.57 | 69 | 1144.41 | 64.93 |  |  |  |
|  | V3 | Placebo | 78 | 983.37 | 35.08 | 76 | 1173.81 | 56.12 | 76 | 1104.25 | 57.20 |  |  |  |
|  |  | PS | 69 | 1019.47 | 57.64 | 69 | 1253.60 | 81.85 | 67 | 1196.69 | 89.57 |  |  |  |
| Arrow flankers (Overall  - %correct) | V1 | Placebo | 77 | 95.60 | 0.80 | 77 | 95.74 | 0.62 | 76 | 94.63 | 0.80 | Tr | 1.86 | 0.18 |
|  |  | PS | 65 | 96.96 | 0.54 | 65 | 95.75 | 0.54 | 68 | 94.71 | 0.88 | Tr x D | 2.61 | 0.11 |
|  | V2 | Placebo | 76 | 96.27 | 0.45 | 77 | 94.95 | 0.66 | 78 | 95.60 | 0.68 |  |  |  |
|  |  | PS | 67 | 96.04 | 0.67 | 68 | 94.52 | 0.83 | 69 | 94.22 | 0.93 |  |  |  |
|  | V3 | Placebo | 78 | 95.83 | 0.67 | 76 | 95.74 | 0.72 | 76 | 95.70 | 0.68 |  |  |  |
|  |  | PS | 69 | 94.62 | 0.91 | 69 | 94.97 | 0.81 | 67 | 95.00 | 0.74 |  |  |  |
| Arrow flankers (Overall RT - ms) | V1 | Placebo | 77 | 846.48 | 31.52 | 77 | 1010.39 | 64.17 | 76 | 937.21 | 43.56 | Tr | 1.25 | 0.27 |
|  |  | PS | 65 | 857.53 | 35.29 | 65 | 900.19 | 36.42 | 68 | 932.47 | 41.71 | Tr x D | 0.03 | 0.87 |
|  | V2 | Placebo | 76 | 838.59 | 27.92 | 77 | 1011.36 | 54.41 | 78 | 967.47 | 42.73 |  |  |  |
|  |  | PS | 67 | 899.66 | 47.76 | 68 | 1081.77 | 59.39 | 69 | 1053.70 | 67.30 |  |  |  |
|  | V3 | Placebo | 78 | 900.32 | 35.48 | 76 | 1085.77 | 52.31 | 76 | 1008.30 | 44.97 |  |  |  |
|  |  | PS | 69 | 910.05 | 38.30 | 69 | 1201.83 | 77.19 | 67 | 1117.53 | 83.60 |  |  |  |
| Digit vigilance (%correct) | V1 | Placebo | 69 | 71.59 | 1.99 | 64 | 68.04 | 2.10 | 64 | 66.14 | 2.48 | Tr | 0.38 | 0.54 |
|  |  | PS | 65 | 69.42 | 2.21 | 58 | 64.99 | 2.28 | 55 | 68.40 | 2.74 | Tr x D | 0.07 | 0.79 |
|  | V2 | Placebo | 69 | 69.83 | 2.40 | 59 | 63.11 | 2.61 | 63 | 62.43 | 2.43 |  |  |  |
|  |  | PS | 62 | 69.58 | 2.12 | 58 | 62.38 | 2.69 | 54 | 59.42 | 2.59 |  |  |  |
|  | V3 | Placebo | 69 | 65.09 | 2.33 | 61 | 58.53 | 2.90 | 55 | 60.49 | 2.60 |  |  |  |
|  |  | PS | 62 | 68.99 | 2.14 | 52 | 57.93 | 2.59 | 52 | 56.93 | 2.57 |  |  |  |
| Digit vigilance (RT - ms) | V1 | Placebo | 69 | 515.72 | 4.46 | 64 | 517.99 | 4.39 | 64 | 517.58 | 4.98 | Tr | 2.18 | 0.14 |
|  |  | PS | 65 | 520.46 | 4.03 | 58 | 518.40 | 4.60 | 55 | 524.15 | 4.46 | Tr x D | 1.01 | 0.32 |
|  | V2 | Placebo | 69 | 521.04 | 4.19 | 59 | 518.88 | 3.73 | 63 | 521.61 | 4.37 |  |  |  |
|  |  | PS | 62 | 516.88 | 3.95 | 58 | 527.83 | 4.87 | 54 | 530.26 | 4.74 |  |  |  |
|  | V3 | Placebo | 69 | 510.79 | 4.07 | 61 | 507.28 | 7.20 | 55 | 514.70 | 6.98 |  |  |  |
|  |  | PS | 62 | 515.18 | 3.84 | 52 | 526.11 | 4.92 | 52 | 530.27 | 4.74 |  |  |  |
| Digit vigilance (false alarms) | V1 | Placebo | 69 | 2.33 | 0.23 | 64 | 2.37 | 0.23 | 64 | 2.17 | 0.23 | Tr | 1.49 | 0.23 |
|  |  | PS | 65 | 1.92 | 0.19 | 58 | 2.48 | 0.22 | 55 | 1.95 | 0.24 | Tr x D | 0.73 | 0.39 |
|  | V2 | Placebo | 69 | 2.09 | 0.22 | 59 | 2.53 | 0.23 | 63 | 2.37 | 0.21 |  |  |  |
|  |  | PS | 62 | 2.37 | 0.20 | 58 | 2.48 | 0.22 | 54 | 3.19 | 0.26 |  |  |  |
|  | V3 | Placebo | 69 | 2.52 | 0.22 | 61 | 2.08 | 0.20 | 55 | 2.40 | 0.23 |  |  |  |
|  |  | PS | 62 | 2.60 | 0.24 | 52 | 2.58 | 0.24 | 52 | 2.50 | 0.23 |  |  |  |
| RVIP (%correct) | V1 | Placebo | 21 | 34.52 | 3.31 | 18 | 31.71 | 4.36 | 16 | 36.46 | 4.12 | Tr | 3.24 | 0.08 |
|  |  | PS | 19 | 34.65 | 4.03 | 13 | 41.99 | 4.91 | 14 | 34.23 | 4.75 | Tr x D | 0.13 | 0.72 |
|  | V2 | Placebo | 20 | 31.87 | 3.66 | 20 | 34.37 | 3.88 | 18 | 33.33 | 3.67 |  |  |  |
|  |  | PS | 25 | 37.83 | 3.36 | 14 | 35.12 | 4.53 | 15 | 34.72 | 3.70 |  |  |  |
|  | V3 | Placebo | 22 | 29.92 | 4.09 | 21 | 33.93 | 3.70 | 20 | 30.21 | 3.58 |  |  |  |
|  |  | PS | 23 | 41.48 | 3.29 | 19 | 33.55 | 3.03 | 16 | 38.54 | 3.79 |  |  |  |
| RVIP (RT - ms) | V1 | Placebo | 21 | 589.50 | 9.63 | 18 | 575.28 | 16.46 | 16 | 577.16 | 14.43 | Tr | 0.00 | 1.00 |
|  |  | PS | 19 | 572.61 | 13.32 | 13 | 553.78 | 17.47 | 14 | 584.92 | 18.08 | Tr x D | 1.85 | 0.18 |
|  | V2 | Placebo | 20 | 589.31 | 15.00 | 20 | 567.00 | 10.54 | 18 | 574.92 | 17.76 |  |  |  |
|  |  | PS | 25 | 583.65 | 12.97 | 14 | 567.35 | 16.89 | 15 | 552.46 | 20.10 |  |  |  |
|  | V3 | Placebo | 22 | 563.05 | 11.07 | 21 | 578.86 | 12.47 | 20 | 563.96 | 17.10 |  |  |  |
|  |  | PS | 23 | 574.89 | 10.20 | 19 | 572.82 | 13.41 | 16 | 566.08 | 12.73 |  |  |  |
| RVIP (false alarms) | V1 | Placebo | 21 | 3.76 | 0.47 | 18 | 3.39 | 0.44 | 16 | 3.12 | 0.61 | Tr | **5.95** | **0.02** |
|  |  | PS | 19 | 4.63 | 0.72 | 13 | 4.31 | 1.22 | 14 | 3.50 | 0.54 | Tr x D | 0.02 | 0.90 |
|  | V2 | Placebo | 20 | 2.90 | 0.58 | 20 | 2.75 | 0.40 | 18 | 2.67 | 0.58 |  |  |  |
|  |  | PS | 25 | 4.80 | 0.59 | 14 | 3.21 | 0.69 | 15 | 4.07 | 0.49 |  |  |  |
|  | V3 | Placebo | 22 | 2.23 | 0.35 | 21 | 2.52 | 0.70 | 20 | 2.25 | 0.48 |  |  |  |
|  |  | PS | 23 | 3.78 | 0.44 | 19 | 2.84 | 0.46 | 16 | 3.38 | 0.52 |  |  |  |
| Stroop (overall %correct) | V1 | Placebo | 79 | 95.32 | 0.71 | 79 | 95.25 | 0.69 | 79 | 94.91 | 0.81 | Tr | 0.00 | 0.98 |
|  |  | PS | 71 | 95.14 | 0.79 | 70 | 95.71 | 0.55 | 71 | 95.14 | 0.84 | Tr x D | 2.91 | 0.09 |
|  | V2 | Placebo | 79 | 94.18 | 0.84 | 78 | 95.42 | 0.69 | 79 | 94.78 | 0.74 |  |  |  |
|  |  | PS | 72 | 94.69 | 1.00 | 70 | 95.89 | 0.55 | 70 | 95.68 | 0.43 |  |  |  |
|  | V3 | Placebo | 79 | 94.84 | 0.70 | 77 | 95.06 | 0.82 | 79 | 95.82 | 0.69 |  |  |  |
|  |  | PS | 72 | 93.85 | 1.05 | 71 | 94.12 | 1.01 | 71 | 95.21 | 0.74 |  |  |  |
| Stroop (overall RT - ms) | V1 | Placebo | 79 | 1015.83 | 27.36 | 79 | 1021.98 | 29.26 | 79 | 1015.56 | 36.85 | Tr | 0.19 | 0.66 |
|  |  | PS | 71 | 1029.40 | 28.90 | 69 | 1035.82 | 33.98 | 71 | 997.69 | 32.70 | Tr x D | 1.10 | 0.30 |
|  | V2 | Placebo | 79 | 1009.94 | 26.51 | 78 | 1051.46 | 35.99 | 79 | 1010.12 | 39.35 |  |  |  |
|  |  | PS | 72 | 1007.89 | 29.72 | 70 | 1057.16 | 35.01 | 70 | 1040.36 | 42.92 |  |  |  |
|  | V3 | Placebo | 79 | 1037.61 | 32.12 | 77 | 1073.00 | 46.25 | 79 | 1017.18 | 39.29 |  |  |  |
|  |  | PS | 72 | 1057.93 | 43.24 | 71 | 1097.26 | 43.36 | 71 | 1115.50 | 71.27 |  |  |  |
| Stroop (congruent %correct) | V1 | Placebo | 79 | 98.29 | 0.40 | 79 | 96.84 | 0.53 | 79 | 96.65 | 0.57 | Tr | 1.32 | 0.25 |
|  |  | PS | 71 | 97.61 | 0.54 | 69 | 97.54 | 0.50 | 71 | 97.75 | 0.50 | Tr x D | 1.28 | 0.26 |
|  | V2 | Placebo | 79 | 97.22 | 0.56 | 78 | 96.41 | 0.66 | 79 | 95.25 | 0.81 |  |  |  |
|  |  | PS | 72 | 97.01 | 0.65 | 70 | 97.29 | 0.54 | 70 | 96.93 | 0.53 |  |  |  |
|  | V3 | Placebo | 79 | 96.20 | 0.56 | 77 | 96.23 | 0.75 | 79 | 96.46 | 0.79 |  |  |  |
|  |  | PS | 72 | 96.60 | 0.58 | 71 | 95.28 | 0.93 | 71 | 96.90 | 0.67 |  |  |  |
| Stroop (congruent RT - ms) | V1 | Placebo | 79 | 949.79 | 26.79 | 79 | 940.05 | 22.52 | 79 | 942.41 | 29.35 | Tr | 0.25 | 0.62 |
|  |  | PS | 71 | 958.70 | 26.45 | 69 | 974.63 | 31.77 | 71 | 945.54 | 32.66 | Tr x D | 2.10 | 0.15 |
|  | V2 | Placebo | 79 | 937.01 | 23.78 | 78 | 1007.74 | 38.64 | 79 | 951.05 | 39.06 |  |  |  |
|  |  | PS | 72 | 935.93 | 25.19 | 70 | 1004.28 | 33.09 | 70 | 974.33 | 36.03 |  |  |  |
|  | V3 | Placebo | 79 | 990.21 | 32.01 | 77 | 1027.60 | 47.44 | 79 | 942.10 | 26.72 |  |  |  |
|  |  | PS | 72 | 1000.18 | 40.33 | 71 | 1059.50 | 44.39 | 71 | 1076.91 | 74.74 |  |  |  |
| Stroop (incongruent %correct) | V1 | Placebo | 79 | 92.34 | 1.31 | 79 | 93.67 | 1.15 | 79 | 93.16 | 1.38 | Tr | 0.04 | 0.84 |
|  |  | PS | 71 | 92.68 | 1.42 | 69 | 93.77 | 0.82 | 71 | 92.54 | 1.44 | Tr x D | 2.35 | 0.13 |
|  | V2 | Placebo | 79 | 91.14 | 1.49 | 78 | 94.42 | 0.88 | 79 | 94.30 | 0.98 |  |  |  |
|  |  | PS | 72 | 92.36 | 1.89 | 70 | 94.50 | 0.75 | 70 | 94.43 | 0.59 |  |  |  |
|  | V3 | Placebo | 79 | 93.48 | 1.04 | 77 | 93.90 | 1.01 | 79 | 95.19 | 0.73 |  |  |  |
|  |  | PS | 72 | 91.11 | 2.00 | 71 | 92.96 | 1.51 | 71 | 93.52 | 1.21 |  |  |  |
| Stroop (incongruent RT - ms) | V1 | Placebo | 79 | 1085.66 | 30.47 | 79 | 1108.82 | 40.55 | 79 | 1090.04 | 51.42 | Tr | 0.06 | 0.81 |
|  |  | PS | 71 | 1103.42 | 34.80 | 69 | 1100.27 | 38.49 | 71 | 1055.79 | 34.81 | Tr x D | 0.27 | 0.60 |
|  | V2 | Placebo | 79 | 1098.53 | 39.10 | 78 | 1095.67 | 36.65 | 79 | 1074.07 | 44.13 |  |  |  |
|  |  | PS | 72 | 1081.44 | 36.60 | 70 | 1110.82 | 40.98 | 70 | 1109.16 | 55.53 |  |  |  |
|  | V3 | Placebo | 79 | 1091.53 | 38.21 | 77 | 1120.59 | 49.26 | 79 | 1094.72 | 60.08 |  |  |  |
|  |  | PS | 72 | 1133.51 | 50.87 | 71 | 1133.79 | 46.41 | 71 | 1153.29 | 70.62 |  |  |  |
| Corsi blocks (span score) | V1 | Placebo | 78 | 5.13 | 0.16 | 75 | 4.66 | 0.18 | 73 | 4.42 | 0.19 | Tr | 2.69 | 0.10 |
|  |  | PS | 70 | 5.28 | 0.14 | 70 | 4.43 | 0.21 | 70 | 4.57 | 0.20 | Tr x D | 0.02 | 0.89 |
|  | V2 | Placebo | 79 | 4.98 | 0.17 | 77 | 4.45 | 0.20 | 77 | 4.44 | 0.19 |  |  |  |
|  |  | PS | 71 | 5.50 | 0.13 | 69 | 4.64 | 0.17 | 68 | 4.62 | 0.20 |  |  |  |
|  | V3 | Placebo | 78 | 4.83 | 0.17 | 75 | 4.16 | 0.19 | 74 | 4.76 | 0.17 |  |  |  |
|  |  | PS | 71 | 5.15 | 0.17 | 64 | 4.58 | 0.23 | 65 | 4.67 | 0.20 |  |  |  |
| Numeric working memory (%correct) | V1 | Placebo | 77 | 86.15 | 1.00 | 77 | 83.56 | 1.25 | 75 | 83.24 | 1.28 | Tr | 1.42 | 0.24 |
|  |  | PS | 71 | 86.87 | 1.15 | 71 | 85.09 | 1.21 | 72 | 84.20 | 1.21 | Tr x D | 3.42 | 0.07 |
|  | V2 | Placebo | 79 | 84.92 | 1.30 | 77 | 82.37 | 1.47 | 75 | 82.50 | 1.57 |  |  |  |
|  |  | PS | 72 | 87.02 | 1.29 | 71 | 84.23 | 1.28 | 69 | 82.38 | 1.48 |  |  |  |
|  | V3 | Placebo | 78 | 84.99 | 1.34 | 72 | 82.48 | 1.55 | 75 | 81.96 | 1.53 |  |  |  |
|  |  | PS | 69 | 89.94 | 0.99 | 71 | 84.15 | 1.42 | 68 | 84.35 | 1.40 |  |  |  |
| Numeric working memory (RT - ms) | V1 | Placebo | 77 | 1256.45 | 49.54 | 77 | 1234.93 | 50.35 | 75 | 1134.50 | 43.04 | Tr | 0.60 | 0.44 |
|  |  | PS | 71 | 1257.71 | 42.95 | 71 | 1190.71 | 45.27 | 72 | 1154.66 | 38.82 | Tr x D | 2.09 | 0.15 |
|  | V2 | Placebo | 79 | 1219.11 | 44.99 | 77 | 1133.16 | 41.54 | 75 | 1119.25 | 44.08 |  |  |  |
|  |  | PS | 72 | 1196.13 | 44.22 | 71 | 1189.10 | 53.68 | 69 | 1136.09 | 51.08 |  |  |  |
|  | V3 | Placebo | 78 | 1192.14 | 40.09 | 72 | 1142.47 | 46.74 | 75 | 1086.13 | 45.30 |  |  |  |
|  |  | PS | 69 | 1199.32 | 46.27 | 71 | 1215.20 | 50.65 | 68 | 1212.79 | 67.11 |  |  |  |
| 2-back (%correct) | V1 | Placebo | 72 | 74.91 | 2.47 | 63 | 80.85 | 2.30 | 65 | 80.92 | 2.19 | Tr | 3.05 | 0.08 |
|  |  | PS | 60 | 80.44 | 2.59 | 54 | 83.58 | 2.76 | 55 | 81.33 | 2.28 | Tr x D | 1.54 | 0.22 |
|  | V2 | Placebo | 74 | 82.34 | 2.06 | 64 | 79.27 | 2.33 | 62 | 80.21 | 2.40 |  |  |  |
|  |  | PS | 61 | 84.92 | 2.27 | 60 | 84.44 | 2.54 | 53 | 85.03 | 1.92 |  |  |  |
|  | V3 | Placebo | 67 | 81.49 | 2.46 | 64 | 78.44 | 2.62 | 61 | 80.87 | 2.26 |  |  |  |
|  |  | PS | 62 | 83.98 | 2.13 | 58 | 81.03 | 2.83 | 55 | 82.30 | 2.52 |  |  |  |
| 2-back (RT - ms) | V1 | Placebo | 72 | 8319.42 | 430.36 | 63 | 7522.20 | 405.03 | 65 | 7073.06 | 256.28 | Tr | 1.31 | 0.26 |
|  |  | PS | 60 | 8508.04 | 706.98 | 54 | 7212.42 | 366.41 | 55 | 7283.00 | 320.89 | Tr x D | 0.06 | 0.81 |
|  | V2 | Placebo | 74 | 7012.83 | 353.17 | 64 | 7307.53 | 308.10 | 62 | 7234.43 | 353.12 |  |  |  |
|  |  | PS | 61 | 6876.67 | 220.97 | 60 | 7897.39 | 1135.76 | 53 | 6664.63 | 264.67 |  |  |  |
|  | V3 | Placebo | 67 | 7760.42 | 587.80 | 64 | 8325.69 | 600.07 | 61 | 6720.70 | 266.28 |  |  |  |
|  |  | PS | 62 | 6798.52 | 186.52 | 58 | 7952.33 | 509.14 | 55 | 7333.17 | 337.18 |  |  |  |
| 2-back (Errors-number) | V1 | Placebo | 72 | 2.43 | 0.31 | 63 | 2.51 | 0.31 | 65 | 2.94 | 0.39 | Tr | 0.84 | 0.36 |
|  |  | PS | 60 | 2.12 | 0.31 | 54 | 2.15 | 0.34 | 55 | 2.40 | 0.33 | Tr x D | 1.06 | 0.31 |
|  | V2 | Placebo | 74 | 3.03 | 0.37 | 64 | 2.47 | 0.32 | 62 | 2.48 | 0.36 |  |  |  |
|  |  | PS | 61 | 2.00 | 0.24 | 60 | 2.57 | 0.36 | 53 | 2.77 | 0.34 |  |  |  |
|  | V3 | Placebo | 67 | 2.03 | 0.31 | 64 | 2.22 | 0.31 | 61 | 2.77 | 0.34 |  |  |  |
|  |  | PS | 62 | 1.89 | 0.29 | 58 | 2.07 | 0.31 | 55 | 2.42 | 0.29 |  |  |  |

The final two columns show effects (F) and associated probabilities (p). Tr=main effect of supplement; Tr x D=supplement x day interaction.

# Table S5. Means (plus SEM) derived from the linear mixed model analysis of COMPASS task outcomes measured during two assessments at each study visit for PP population.

|  |  |  | Assessment 1 | | | Assessment 3 | | |  |  |  |
| --- | --- | --- | --- | --- | --- | --- | --- | --- | --- | --- | --- |
|  | visit | Treatment | N | Mean | SEM | N | Mean | SEM | Analysis | F | p |
| Immediate word recall (%correct) | V1 | Placebo | 79 | 30.97 | 1.13 | 79 | 27.93 | 1.16 | Tr | 0.57 | 0.452 |
|  |  | PS | 71 | 29.53 | 1.46 | 72 | 28.61 | 1.34 | Tr x D | 0.02 | 0.882 |
|  | V2 | Placebo | 79 | 33.12 | 1.05 | 79 | 27.64 | 1.31 |  |  |  |
|  |  | PS | 72 | 33.06 | 1.25 | 71 | 28.31 | 1.35 |  |  |  |
|  | V3 | Placebo | 79 | 33.04 | 1.26 | 79 | 27.81 | 1.39 |  |  |  |
|  |  | PS | 72 | 33.52 | 1.46 | 72 | 28.52 | 1.45 |  |  |  |
| Delayed word recall (%correct) | V1 | Placebo | 79 | 21.18 | 1.19 | 79 | 12.03 | 1.34 | Tr | 0.01 | 0.941 |
|  |  | PS | 71 | 19.20 | 1.34 | 71 | 11.13 | 1.32 | Tr x D | 0.01 | 0.942 |
|  | V2 | Placebo | 79 | 26.03 | 1.20 | 79 | 14.35 | 1.33 |  |  |  |
|  |  | PS | 72 | 25.09 | 1.37 | 70 | 13.76 | 1.33 |  |  |  |
|  | V3 | Placebo | 79 | 28.23 | 1.31 | 79 | 13.29 | 1.33 |  |  |  |
|  |  | PS | 72 | 25.83 | 1.56 | 72 | 14.12 | 1.46 |  |  |  |
| Delayed picture recognition (%correct) | V1 | Placebo | 79 | 89.29 | 0.92 | 78 | 84.24 | 1.34 | Tr | 0.11 | 0.742 |
|  |  | PS | 72 | 88.02 | 1.10 | 72 | 82.52 | 1.46 | Tr x D | 1.05 | 0.307 |
|  | V2 | Placebo | 79 | 91.03 | 0.97 | 78 | 82.75 | 1.45 |  |  |  |
|  |  | PS | 72 | 88.89 | 1.17 | 69 | 82.79 | 1.64 |  |  |  |
|  | V3 | Placebo | 78 | 86.65 | 1.22 | 78 | 81.62 | 1.38 |  |  |  |
|  |  | PS | 72 | 87.62 | 1.34 | 71 | 80.69 | 1.37 |  |  |  |
| Delayed picture recognition RT - ms) | V1 | Placebo | 79 | 1040.03 | 21.58 | 78 | 992.10 | 22.63 | Tr | 1.45 | 0.230 |
|  |  | PS | 72 | 1060.02 | 26.71 | 72 | 1055.51 | 29.97 | Tr x D | 0.79 | 0.375 |
|  | V2 | Placebo | 79 | 1026.55 | 23.91 | 78 | 998.04 | 24.03 |  |  |  |
|  |  | PS | 72 | 996.10 | 25.94 | 69 | 1031.29 | 32.52 |  |  |  |
|  | V3 | Placebo | 78 | 1022.98 | 25.60 | 78 | 1028.62 | 29.35 |  |  |  |
|  |  | PS | 72 | 1009.19 | 26.36 | 71 | 993.78 | 28.28 |  |  |  |
| Delayed word recognition (%correct) | V1 | Placebo | 77 | 79.33 | 1.03 | 76 | 74.78 | 1.34 | Tr | 1.6 | 0.208 |
|  |  | PS | 71 | 77.23 | 1.30 | 67 | 73.94 | 1.36 | Tr x D | 0.15 | 0.695 |
|  | V2 | Placebo | 78 | 82.00 | 0.98 | 77 | 74.08 | 1.29 |  |  |  |
|  |  | PS | 72 | 82.29 | 1.14 | 69 | 75.30 | 1.25 |  |  |  |
|  | V3 | Placebo | 77 | 80.57 | 1.17 | 79 | 72.63 | 1.20 |  |  |  |
|  |  | PS | 71 | 81.75 | 1.30 | 69 | 74.03 | 1.35 |  |  |  |
| Delayed word recognition RT - ms) | V1 | Placebo | 77 | 1195.46 | 41.76 | 76 | 1085.54 | 44.14 | Tr | 0.1 | 0.752 |
|  |  | PS | 71 | 1164.12 | 34.04 | 67 | 1093.53 | 40.48 | Tr x D | 2.49 | 0.116 |
|  | V2 | Placebo | 78 | 1129.76 | 48.57 | 77 | 1063.12 | 35.61 |  |  |  |
|  |  | PS | 72 | 1099.40 | 37.64 | 69 | 1054.81 | 31.06 |  |  |  |
|  | V3 | Placebo | 77 | 1078.46 | 45.51 | 79 | 1032.56 | 26.90 |  |  |  |
|  |  | PS | 71 | 1082.47 | 33.44 | 69 | 1077.81 | 35.85 |  |  |  |

The final two columns show effects (F) and associated probabilities (p). Tr=main effect of supplement; Tr x D=supplement x day interaction.

# Table S6. Means (plus SEM) derived from the linear mixed model analysis of mood visual analogue scales measured during two assessments at each study visit for PP population.

|  |  |  | Assessment 1 | | | Assessment 3 | | |  |  |  |
| --- | --- | --- | --- | --- | --- | --- | --- | --- | --- | --- | --- |
|  | visit | Treatment | N | Mean | SEM | N | Mean | SEM | Analysis | F | p |
| Relaxed | V1 | Placebo | 79 | 66.04 | 2.28 | 79 | 69.42 | 2.55 | Tr | 0.90 | 0.344 |
|  |  | PS | 72 | 65.19 | 1.98 | 72 | 68.07 | 2.65 | Tr x D | 0.24 | 0.627 |
|  | V2 | Placebo | 79 | 64.53 | 2.43 | 79 | 64.37 | 2.67 |  |  |  |
|  |  | PS | 72 | 64.72 | 2.25 | 72 | 65.88 | 2.81 |  |  |  |
|  | V3 | Placebo | 79 | 62.53 | 2.48 | 79 | 62.81 | 2.72 |  |  |  |
|  |  | PS | 72 | 62.69 | 2.03 | 72 | 66.63 | 2.46 |  |  |  |
| Alert | V1 | Placebo | 79 | 46.91 | 2.84 | 79 | 46.54 | 3.25 | Tr | 1.03 | 0.312 |
|  |  | PS | 72 | 42.47 | 2.88 | 72 | 41.85 | 3.32 | Tr x D | 0.02 | 0.893 |
|  | V2 | Placebo | 79 | 47.34 | 2.89 | 79 | 47.44 | 3.56 |  |  |  |
|  |  | PS | 72 | 42.36 | 2.93 | 72 | 42.57 | 3.14 |  |  |  |
|  | V3 | Placebo | 79 | 46.46 | 3.00 | 79 | 49.38 | 3.28 |  |  |  |
|  |  | PS | 72 | 42.40 | 3.13 | 72 | 42.89 | 3.25 |  |  |  |
| Jittery | V1 | Placebo | 79 | 23.68 | 2.52 | 79 | 26.68 | 2.71 | Tr | 0.04 | 0.835 |
|  |  | PS | 72 | 26.10 | 2.75 | 72 | 22.74 | 2.93 | Tr x D | 0.01 | 0.910 |
|  | V2 | Placebo | 79 | 23.80 | 2.57 | 79 | 20.90 | 2.40 |  |  |  |
|  |  | PS | 72 | 21.68 | 2.62 | 72 | 23.60 | 2.81 |  |  |  |
|  | V3 | Placebo | 79 | 25.76 | 2.78 | 79 | 25.41 | 2.51 |  |  |  |
|  |  | PS | 72 | 27.72 | 2.89 | 72 | 23.44 | 2.98 |  |  |  |
| Tired | V1 | Placebo | 79 | 34.16 | 2.93 | 79 | 41.59 | 3.21 | Tr | 1.10 | 0.296 |
|  |  | PS | 72 | 39.08 | 2.91 | 72 | 42.96 | 3.07 | Tr x D | 0.11 | 0.741 |
|  | V2 | Placebo | 79 | 41.68 | 3.03 | 79 | 38.06 | 2.99 |  |  |  |
|  |  | PS | 72 | 37.13 | 2.78 | 72 | 39.19 | 3.18 |  |  |  |
|  | V3 | Placebo | 79 | 42.35 | 2.95 | 79 | 43.11 | 2.77 |  |  |  |
|  |  | PS | 72 | 42.88 | 2.87 | 72 | 40.93 | 3.10 |  |  |  |
| Tense | V1 | Placebo | 79 | 27.38 | 2.26 | 79 | 28.86 | 2.60 | Tr | 0.16 | 0.695 |
|  |  | PS | 72 | 25.03 | 2.34 | 72 | 24.19 | 2.56 | Tr x D | 0.31 | 0.578 |
|  | V2 | Placebo | 79 | 24.13 | 2.37 | 79 | 26.25 | 2.42 |  |  |  |
|  |  | PS | 72 | 23.17 | 2.30 | 72 | 22.13 | 2.56 |  |  |  |
|  | V3 | Placebo | 79 | 26.89 | 2.50 | 79 | 25.01 | 2.47 |  |  |  |
|  |  | PS | 72 | 23.92 | 2.47 | 72 | 25.53 | 2.34 |  |  |  |
| Headache | V1 | PLA | 79 | 9.09 | 1.91 | 79 | 19.37 | 2.69 | Tr | 0.37 | 0.543 |
|  |  | PS | 72 | 9.65 | 1.90 | 72 | 15.25 | 2.48 | Tr x D | 0.74 | 0.392 |
|  | V2 | Placebo | 79 | 12.06 | 2.26 | 79 | 16.38 | 2.62 |  |  |  |
|  |  | PS | 72 | 10.35 | 1.81 | 72 | 12.24 | 1.90 |  |  |  |
|  | V3 | Placebo | 79 | 12.46 | 1.91 | 79 | 17.44 | 2.37 |  |  |  |
|  |  | PS | 72 | 12.19 | 2.10 | 72 | 15.81 | 2.59 |  |  |  |
| Overall mood | V1 | Placebo | 79 | 75.77 | 1.90 | 79 | 74.86 | 2.27 | Tr | 0.15 | 0.697 |
|  |  | PS | 72 | 75.15 | 2.01 | 72 | 73.94 | 2.36 | Tr x D | 0.15 | 0.696 |
|  | V2 | Placebo | 79 | 73.72 | 2.15 | 79 | 75.84 | 2.41 |  |  |  |
|  |  | PS | 72 | 72.56 | 2.31 | 72 | 74.90 | 2.34 |  |  |  |
|  | V3 | Placebo | 79 | 71.82 | 2.24 | 79 | 72.09 | 2.27 |  |  |  |
|  |  | PS | 72 | 71.63 | 2.29 | 72 | 71.89 | 2.20 |  |  |  |
| Mental fatigue | V1 | Placebo | 79 | 28.59 | 2.53 | 79 | 34.32 | 3.05 | Tr | 0.53 | 0.466 |
|  |  | PS | 72 | 22.94 | 2.53 | 72 | 28.01 | 2.83 | Tr x D | 0.01 | 0.912 |
|  | V2 | Placebo | 79 | 26.42 | 2.58 | 79 | 34.24 | 3.14 |  |  |  |
|  |  | PS | 72 | 24.50 | 2.55 | 72 | 33.51 | 2.85 |  |  |  |
|  | V3 | Placebo | 79 | 24.89 | 2.63 | 79 | 36.09 | 3.06 |  |  |  |
|  |  | PS | 72 | 24.00 | 2.29 | 72 | 33.78 | 2.98 |  |  |  |
| Performing | V1 | Placebo | 79 | 26.04 | 1.94 | 79 | 27.27 | 2.34 | Tr | 0.33 | 0.568 |
|  |  | PS | 72 | 28.60 | 1.98 | 72 | 30.46 | 2.22 | Tr x D | 0.91 | 0.340 |
|  | V2 | Placebo | 79 | 28.65 | 1.98 | 79 | 26.72 | 2.14 |  |  |  |
|  |  | PS | 72 | 28.79 | 2.09 | 72 | 28.90 | 2.38 |  |  |  |
|  | V3 | Placebo | 79 | 28.08 | 2.39 | 79 | 28.48 | 2.44 |  |  |  |
|  |  | PS | 72 | 31.71 | 2.32 | 72 | 31.82 | 2.48 |  |  |  |
| Happy/sad | V1 | Placebo | 79 | 20.24 | 1.88 | 79 | 21.42 | 2.14 | Tr | 1.27 | 0.262 |
|  |  | PS | 72 | 18.36 | 2.14 | 72 | 19.53 | 2.20 | Tr x D | 1.16 | 0.282 |
|  | V2 | Placebo | 79 | 19.96 | 2.17 | 79 | 21.56 | 2.08 |  |  |  |
|  |  | PS | 72 | 20.19 | 2.19 | 72 | 22.36 | 2.26 |  |  |  |
|  | V3 | Placebo | 79 | 21.28 | 2.11 | 79 | 20.92 | 2.25 |  |  |  |
|  |  | PS | 72 | 22.99 | 2.37 | 72 | 25.21 | 2.38 |  |  |  |
| Stressed/calm | V1 | Placebo | 79 | 72.32 | 2.55 | 79 | 69.99 | 2.70 | Tr | 1.88 | 0.173 |
|  |  | PS | 72 | 74.22 | 2.58 | 72 | 73.90 | 2.61 | Tr x D | 0.26 | 0.612 |
|  | V2 | Placebo | 79 | 71.70 | 2.66 | 79 | 71.92 | 2.88 |  |  |  |
|  |  | PS | 72 | 70.42 | 2.57 | 72 | 70.07 | 2.65 |  |  |  |
|  | V3 | Placebo | 79 | 71.56 | 2.52 | 79 | 72.10 | 2.55 |  |  |  |
|  |  | PS | 72 | 69.00 | 2.57 | 72 | 69.25 | 2.80 |  |  |  |

The final two columns show effects (F) and associated probabilities (p). Tr=main effect of supplement; Tr x D=supplement x day interaction.

# Table S7. Means (plus SEM) derived from the linear mixed model analysis of parent visual analogue scales for PP population.

|  | visit | Arm | N | Mean | SEM | Analysis | F | p |
| --- | --- | --- | --- | --- | --- | --- | --- | --- |
| CSHQ  total score | V1 | Placebo | 75 | 31.40 | 0.69 | Tr | 0.03 | 0.856 |
|  |  | PS | 70 | 30.61 | 0.58 | Tr x D | 0.19 | 0.661 |
|  | V2 | Placebo | 79 | 30.91 | 0.60 |  |  |  |
|  |  | PS | 72 | 30.53 | 0.58 |  |  |  |
|  | V3 | Placebo | 79 | 30.63 | 0.61 |  |  |  |
|  |  | PS | 72 | 30.22 | 0.59 |  |  |  |
| CSSR total score | V1 | Placebo | 79 | 32.31 | 0.57 | Tr | 0.00 | 0.951 |
|  |  | PS | 72 | 32.24 | 0.58 | Tr x D | 0.60 | 0.441 |
|  | V2 | Placebo | 79 | 31.33 | 0.39 |  |  |  |
|  |  | PS | 72 | 31.50 | 0.41 |  |  |  |
|  | V3 | Placebo | 78 | 31.69 | 0.39 |  |  |  |
|  |  | PS | 72 | 31.45 | 0.41 |  |  |  |

The final two columns show effects (F) and associated probabilities (p). Tr=main effect of supplement; Tr x D=supplement x day interaction.

# Table S8. Means (plus SEM) derived from the linear mixed model analysis of sleep actigraphy for PP population.

|  |  |  | Night 1 | | | Night 2 | | | Night 3 | | | Night 4 | | | Night 5 | | | Night 6 | | | Night 7 | | | |  |  | |
| --- | --- | --- | --- | --- | --- | --- | --- | --- | --- | --- | --- | --- | --- | --- | --- | --- | --- | --- | --- | --- | --- | --- | --- | --- | --- | --- | --- |
|  | Visit | Arm | N | Mean | SEM | N | Mean | SEM | N | Mean | SEM | N | Mean | SEM | N | Mean | SEM | N | Mean | SEM | N | Mean | SEM | Analysis | F | p |  |
| Sleep onset | V1 | Placebo | 22 | 11.09 | 1.09 | 24 | 13.08 | 1.87 | 24 | 14.83 | 2.25 | 23 | 15.22 | 3.49 | 24 | 12.67 | 1.82 | 24 | 12.67 | 1.91 | 24 | 13.38 | 2.55 | Tr | 0.22 | 0.643 |  |
|  |  | PS | 19 | 13 | 2.24 | 19 | 12.42 | 2.16 | 18 | 14 | 2.53 | 19 | 12.16 | 1.49 | 19 | 11.32 | 2.22 | 19 | 9.26 | 2.49 | 19 | 9.11 | 1.31 |  |  |  |  |
|  | V3 | Placebo | 23 | 11.91 | 2.46 | 23 | 13.91 | 2.12 | 24 | 15.21 | 2.18 | 22 | 13.18 | 2.17 | 24 | 11.33 | 1.62 | 22 | 10 | 1.63 | 22 | 12.82 | 2.46 |  |  |  |  |
|  |  | PS | 16 | 12.31 | 2.39 | 19 | 13.11 | 1.91 | 19 | 9.42 | 1.42 | 19 | 12 | 1.62 | 19 | 8.21 | 1.54 | 18 | 12.83 | 2.23 | 18 | 14.39 | 2.21 |  |  |  |  |
| Total sleep time | V1 | Placebo | 22 | 486.91 | 9.91 | 24 | 488.83 | 9.52 | 24 | 476.79 | 10.14 | 23 | 498 | 10.97 | 24 | 483.29 | 8.92 | 24 | 487.63 | 9.78 | 24 | 463.71 | 10.81 | Tr | 0.06 | 0.808 |  |
|  |  | PS | 19 | 491.11 | 10.84 | 19 | 492.47 | 11.04 | 18 | 491.17 | 10.68 | 19 | 496.05 | 11.99 | 19 | 483.74 | 11.37 | 19 | 484.74 | 10.86 | 19 | 457.16 | 10.74 |  |  |  |  |
|  | V3 | Placebo | 23 | 489.57 | 14.24 | 23 | 499.26 | 9.87 | 24 | 480.25 | 10.55 | 22 | 470.59 | 8.1 | 24 | 475.37 | 8.46 | 22 | 458.91 | 11.89 | 22 | 452.23 | 9.89 |  |  |  |  |
|  |  | PS | 16 | 478.81 | 13.39 | 19 | 483.47 | 10.99 | 19 | 470.42 | 10.93 | 19 | 477.58 | 14.78 | 19 | 498.74 | 16.26 | 18 | 462.83 | 11.89 | 18 | 457.56 | 11.18 |  |  |  |  |
| Wake after sleep onset | V1 | Placebo | 22 | 78.09 | 7.5 | 24 | 79.13 | 7.02 | 24 | 86.67 | 7.04 | 23 | 75.13 | 6.67 | 24 | 84.54 | 8.45 | 24 | 82.5 | 6.82 | 24 | 77.21 | 6.01 | Tr | 1.51 | 0.227 |  |
|  |  | PS | 19 | 64.21 | 7.95 | 19 | 65.68 | 8.09 | 18 | 64 | 6.44 | 19 | 65.68 | 6.14 | 19 | 65.58 | 8.7 | 19 | 71.47 | 6.48 | 19 | 73.63 | 7.83 |  |  |  |  |
|  | V3 | Placebo | 23 | 74.35 | 9.15 | 23 | 75 | 6.34 | 24 | 77.08 | 7.39 | 22 | 82.73 | 7.47 | 24 | 79.33 | 8.07 | 22 | 79.73 | 6.59 | 22 | 74.5 | 7.04 |  |  |  |  |
|  |  | PS | 16 | 71.25 | 10.44 | 19 | 63.74 | 5.6 | 19 | 75.42 | 6.75 | 19 | 70.26 | 9.5 | 19 | 64.89 | 8.39 | 18 | 68.56 | 7.99 | 18 | 64.22 | 8.65 |  |  |  |  |
| Awakenings | V1 | Placebo | 22 | 23.68 | 1.06 | 24 | 25.46 | 1.66 | 24 | 25.71 | 1.52 | 23 | 24.78 | 1.84 | 24 | 25.71 | 1.52 | 24 | 27.33 | 1.62 | 24 | 24.54 | 1.27 | Tr | 3.61 | 0.066 |  |
|  |  | PS | 19 | 21.68 | 1.7 | 19 | 22.37 | 1.73 | 18 | 23.44 | 1.75 | 19 | 24.21 | 1.67 | 19 | 23.26 | 1.92 | 19 | 26.05 | 1.29 | 19 | 21.95 | 1.56 |  |  |  |  |
|  | V3 | Placebo | 23 | 24.13 | 1.72 | 23 | 26.17 | 1.52 | 24 | 24.42 | 1.45 | 22 | 25.09 | 1.44 | 24 | 25.96 | 1.69 | 22 | 24.5 | 1.37 | 22 | 23.86 | 1.74 |  |  |  |  |
|  |  | PS | 16 | 24.25 | 1.93 | 19 | 20.84 | 1.03 | 19 | 23.42 | 1.19 | 19 | 23.37 | 1.8 | 19 | 21.68 | 2.08 | 18 | 23.11 | 1.72 | 18 | 20.94 | 1.35 |  |  |  |  |
| Average awakenings | V1 | Placebo | 22 | 3.24 | 0.25 | 24 | 3.12 | 0.22 | 24 | 3.47 | 0.31 | 23 | 3.18 | 0.31 | 24 | 3.21 | 0.25 | 24 | 3.01 | 0.18 | 24 | 3.13 | 0.19 | Tr | 0.13 | 0.717 |  |
|  |  | PS | 19 | 2.86 | 0.24 | 19 | 2.74 | 0.2 | 18 | 2.77 | 0.22 | 19 | 2.72 | 0.17 | 19 | 2.72 | 0.18 | 19 | 2.71 | 0.18 | 19 | 3.36 | 0.28 |  |  |  |  |
|  | V3 | Placebo | 23 | 2.99 | 0.26 | 23 | 2.85 | 0.17 | 24 | 3.1 | 0.22 | 22 | 3.27 | 0.22 | 24 | 2.99 | 0.21 | 22 | 3.28 | 0.25 | 22 | 3.1 | 0.18 |  |  |  |  |
|  |  | PS | 16 | 2.88 | 0.31 | 19 | 3.06 | 0.26 | 19 | 3.17 | 0.19 | 19 | 2.9 | 0.31 | 19 | 2.97 | 0.19 | 18 | 2.97 | 0.27 | 18 | 2.87 | 0.26 |  |  |  |  |
| Efficiency | V1 | Placebo | 22 | 84.65 | 1.29 | 24 | 84.24 | 1.13 | 24 | 82.54 | 1.21 | 23 | 84.73 | 1.26 | 24 | 83.42 | 1.4 | 24 | 83.92 | 1.06 | 24 | 83.76 | 1.14 | Tr | 1.1 | 0.302 |  |
|  |  | PS | 19 | 86.42 | 1.45 | 19 | 86.29 | 1.46 | 18 | 86.25 | 1.24 | 19 | 86.29 | 1.16 | 19 | 86.35 | 1.57 | 19 | 85.75 | 1.26 | 19 | 84.79 | 1.41 |  |  |  |  |
|  | V3 | Placebo | 23 | 85.13 | 1.34 | 23 | 84.95 | 0.97 | 24 | 83.92 | 1.3 | 22 | 83.29 | 1.25 | 24 | 84.22 | 1.28 | 22 | 83.64 | 1.22 | 22 | 83.98 | 1.29 |  |  |  |  |
|  |  | PS | 16 | 85.14 | 1.74 | 19 | 86.25 | 1.03 | 19 | 84.75 | 1.16 | 19 | 85.35 | 1.62 | 19 | 87.11 | 1.38 | 18 | 85.02 | 1.37 | 18 | 85.47 | 1.49 |  |  |  |  |
| Total time in bed | V1 | Placebo | 22 | 576.09 | 10.44 | 24 | 581.04 | 10.42 | 24 | 578.29 | 10.49 | 23 | 588.35 | 11.21 | 24 | 580.5 | 8.63 | 24 | 582.79 | 12.91 | 24 | 554.29 | 12.16 | Tr | 1.38 | 0.247 |  |
|  |  | PS | 19 | 568.32 | 8.06 | 19 | 570.58 | 7.94 | 18 | 569.17 | 7.77 | 19 | 573.89 | 8.06 | 19 | 560.63 | 9.76 | 19 | 565.47 | 9.82 | 19 | 539.89 | 11.21 |  |  |  |  |
|  | V3 | Placebo | 23 | 575.83 | 15.22 | 23 | 588.17 | 10.89 | 24 | 572.54 | 9.85 | 22 | 566.5 | 10.36 | 24 | 566.04 | 10.07 | 22 | 548.64 | 12.01 | 22 | 539.55 | 11.06 |  |  |  |  |
|  |  | PS | 16 | 562.38 | 10.33 | 19 | 560.32 | 10.27 | 19 | 555.26 | 10.58 | 19 | 559.84 | 13.93 | 19 | 571.84 | 14.17 | 18 | 544.22 | 10.32 | 18 | 536.17 | 11.5 |  |  |  |  |

The final two columns show effects (F) and associated probabilities (p). Tr=main effect of supplement.
